# Novel thiazole derivatives as effective anti-cancer agents against human osteosarcoma cell line (SaOS-2): Microwave-assisted one-pot three-component synthesis, in vitro anti-cancer studies, and in silico investigations

**DOI:** 10.1371/journal.pone.0328221

**Published:** 2025-08-26

**Authors:** Arif Mermer, Aydın Tahmasebifar, Sami A. Al-Hussain, Gizem Tatar Yılmaz, Zeynep Dilan Turgut, Aqsa Mushtaq, Ameer Fawad Zahoor, Ali Irfan, Magdi E. A. Zaki

**Affiliations:** 1 Department of Biotechnology, University of Health Sciences, Istanbul, Türkiye; 2 Experimental Medicine Application & Research Center, Validebağ Research Park, University of Health Sciences, Istanbul, Türkiye; 3 Department of Tissue Engineering, University of Health Sciences, Istanbul, Türkiye; 4 Department of Biomaterials, University of Health Sciences Turkey, Istanbul, Türkiye; 5 Department of Chemistry, College of Science, Imam Mohammad Ibn Saud Islamic University (IMSIU), Riyadh, Saudi Arabia; 6 Department of Biostatistics and Medical Informatics, Faculty of Medicine, Karadeniz Technical University, Trabzon, Türkiye; 7 Department of Bioinformatics, Karadeniz Technical University, Institute of Health Sciences, Trabzon, Türkiye; 8 Yılmaz Bilişim R&D Consulting Software Engineering and Services Trade Limited Company, Trabzon, Turkey; 9 Department of Chemistry, Government College University Faisalabad, Faisalabad, Pakistan; Vignan Pharmacy College, INDIA

## Abstract

The current research involves the preparation of novel thiazole derivatives, which was carried out via one-pot three-component reaction utilizing conventional and microwave irradiation (MW) methods. The MW method reduced the reaction time and allowed the reactions to be carried out with higher yields. ^1^H NMR, ^13^C NMR, EI-MS and FT-IR techniques were employed for the characterization of synthesized compounds. All compounds exhibited anti-cancer activity ranging from 0.190 to 0.273 µg/mL against SaOS-2 cell line and the best activity was shown by compound **4i** which exhibited IC_50_ value = 0.190 ± 0.045 µg/mL. It is evident that the concentration of these compounds is a critical factor in determining their biological efficacy. This dose-dependent relationship highlights the importance of optimizing compound concentrations to achieve maximal therapeutic benefits while minimizing potential side effects. Moreover, these findings demonstrated the potential of thiazole derivatives as promising candidates for anti-cancer drug development and warrant further investigation into their mechanisms of action and therapeutic applications. Molecular modelling was utilized to predict potential interactions of the synthesized compounds that exhibited inhibitory effects. The analyses revealed that compound **4i** exhibited strong inhibitory effects against EGFR (docking score: −6.434, MM-GBSA energy: −53.40 kcal/mol) in *in silico* studies.

## 1. Introduction

Cancer has emerged as a prominent global public health issue and is determined to be 2^nd^ major factor of deaths in both developed and developing nations [1]. Among various cancer types, surgery, radiotherapy, and chemotherapy are the primary treatments for osteosarcoma-a malignant and invasive tumor originating from bone marrow mesenchymal cells, commonly seen in children and adolescents, with other cancer therapies [2–12]. Despite chemotherapy being widely used in cancer treatment, its side effects have limited its overall application.

Doxorubicin (DOX), also known as Adriamycin, is a chemotherapy drug with broad anti-tumor and anti-cancer properties, commonly used to treat osteosarcoma and cancers in pediatric or adolescent patients [13]. However, a major challenge in pharmaceutical chemistry is its dose-dependent side effects, particularly cardio- and hepatotoxicity, which are linked to oxidative stress, the generation of ROS (reactive oxygen species), DNA damage and the development of multi-drug resistance (MDR) [14–16]. The overall toxicity associated with DOX, as well as conventional chemotherapeutic agents, remains a critical obstacle in cancer treatment. As a result, the conceptualization and development of novel drugs with lower toxicity and a broader therapeutic spectrum has become a key priority for medicinal chemists worldwide [17].

**Fig 1:**
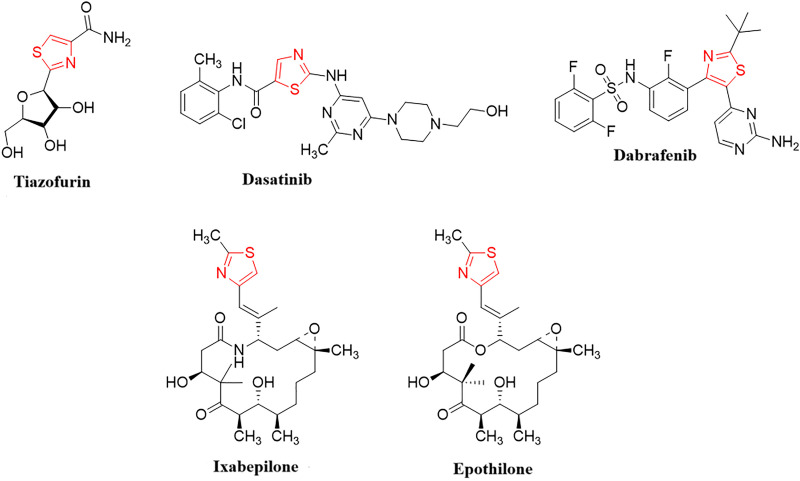
FDA-approved thiazole-containing anti-cancer drugs^34-38.^

Azoles, which constitute a valuable class of heterocycles, play crucial roles in drug design and discovery studies in current medicinal chemistry due to their broad ranging applications [18–20]. Their use is being investigated in different fields of medicine, especially as potential anti-cancer drugs. In recent years, thiazole, a five-membered sulfur- and nitrogen-containing heterocyclic ligand, has attracted great interest due to its effective biological properties including anti-bacterial [21–23], anti-fungal [24], anti-tubercular activity [25,26], anti-viral [27], anti-inflammatory [28], anti-diabetic [29], anti-convulsant [30], anti-oxidant activities [31], and anti-cancer activities [32,33], etc. Furthermore, thiazole-containing compounds have also been found to be structural constituent of clinically available anti-cancer drugs, such as thiazofurin, dasatinib, dabrafenib, ixabepilone, and epothilone (Fig 1) [34–38].

A variety of techniques have been established for the synthesis of iminothiazolines. The earliest of these is the Hantzsch condensation, which involves the synthesis of the 2-amino thiazole unit using an α-haloketone and thiourea [39]. Furthermore, thiazole-2-imines have been synthesised as a result of reaction between isothiocyanates, aromatic α-bromoketones and substituted primary amines [40]. Another pathway involved the reaction between N-propargylanilines and isothiocyanates [41]. Some protocols employ *N*,*N*’-dialkylthiourea and α-bromoketones (in-situ generated) in a one-pot process; however, this approach is constrained to symmetric thioureas and a limited range of ketones [42]. A three-component reaction involving phenacyl bromide or a 2-chloro-1,3-dicarbonyl compound, an amine, and a phenyl isothiocyanate has also been demonstrated to yield thiazole-2-imines [43]. Zhou et al. documented a trypsin-catalysed multicomponent reaction to produce thiazole-2-imines, while another study utilized a conventional one-pot three-component approach with phenacyl bromide, isothiocyanate and amine [44]. While these methods are generally accepted for synthesising thiazole-2-imines, they have several drawbacks, including the use of harsh reaction conditions, prolonged reaction duration and low yields.

In this study, we aimed to enhance the reaction yield within reduced reaction time by using the microwave irradiation method (one of the green chemistry techniques) in order to overcome the above-mentioned disadvantages in the synthesis. At the same time, considering the biological activity spectra of thiazoles, it was thought that the synthesized compounds might have potential anti-cancer activity. Therefore, we synthesized novel thiazole-2-imine derivatives and evaluated their *in vitro* anti-cancer effect against the Saos-2 cell line. Additionally, in silico molecular docking calculations were conducted to elucidate the interactions between the active site of a target protein and the synthesized compounds.

## 2. Results and discussion

### 2.1 Chemistry

A regioselective approach involving one-pot three-component reaction was carried out to obtain thiazol-2-imine derivatives. All synthesized compounds were achieved in excellent yields in short reaction times without further purification methods. Both spectroscopic and spectrometric techniques were utilized to confirm the structures of the synthesized compounds. Considering the FTIR data, the absence of peaks belonging to both the NH_2_ group in the aniline compounds and the carbonyl group in the phenacyl bromide derivative supported the formation of the structures. In particular, in the ^1^H NMR spectrum, the C-5 proton of the thiazole ring was found to resonate in the range of 6.45-6.71 ppm, indicating that ring closure had occurred. Besides, another important evidence is the data obtained from ^13^C NMR spectra where the thiazole C-5 carbon peak was observed in the range of 98.05 to 108.91 ppm. The C-2 and C-4 carbon peaks of the thiazole ring resonated at 157.08-162.63 ppm and 150.41-157.99 ppm, respectively. Although there is a possibility of formation of two products as *E* and *Z* isomers as a result of the reaction, a single isomer formation was identified in the ^1^H NMR spectra of the synthesized compounds. Furthermore, the formation of the *E* isomer is not suitable as the aromatic rings in the aniline and phenyl isothiocyanate groups are very likely to introduce steric hindrance, revealing the instability of the compound. However, in the case of *Z*-isomer these aromatic groups are likely to be free of steric hindrance [44]. Molecular ions peaks of the synthesized compounds were dedicated as [M]^+^ , [M + 1]^+^ or [M + 2]^+^ in the mass spectra.

**Fig 2:**
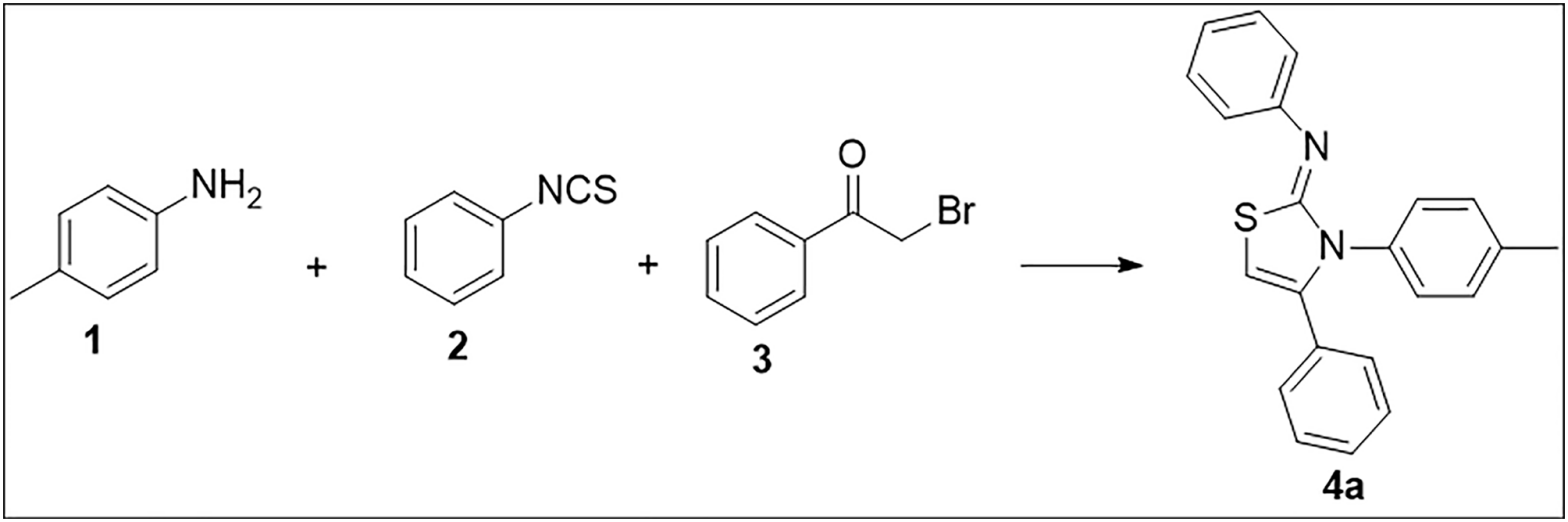
Optimization of model reaction.

Novel thiazole-imine hybrids **4(a-j)** were synthesized through a “one-pot” multicomponent reaction. In this process, two types of amines were reacted with substituted aryl isothiocyanate in ethanol, forming a intermediate (thiosemicarbazide) within 15 minutes. Subsequently, a base and substituted or unsubstituted phenacyl bromide were included to the same reaction mixture, and progress of the reaction was monitored using TLC to obtain the target compounds. Initially, we focused our attention on optimizing the conditions for the model reaction in the conventional method using toluidine, phenyl isothiocyanate, and phenacyl bromide in the presence of different bases such as KOH, NaOH, K_2_CO_3_, NaHCO_3_ and TEA (Fig 2). First, the trial reaction was set up with KOH in the presence of methanol as a solvent, and compound **4a** was synthesized with 61% yield (Table 1, Entry 1). The subsequent investigation of other inorganic bases containing NaOH, NaHCO_3_, and K_2_CO_3_ pointed out that K_2_CO_3_ was the most suitable base for the transformation with a yield of 64%. When the reaction was tried with an organic base, triethyl amine (TEA), it was inferred that the reaction yield (77%) was increased. For a better comparison, an attempt was also made with K_2_CO_3_ in ethanol, but a higher yield was obtained from the reaction using TEA. After determining the base, we decided to use ethanol instead of methanol to see if the solvent had an effect on the yield. Further, changing the reaction temperature led an obvious decrease in yields. Three more solvents were chosen to screen the solvent effects on the reaction, and none of them were as effective on the reaction as ethanol or methanol (Entries 11–13). After specifying the solvent and base, several attempts were made to optimize the reaction time. As a result, it was found that the best conditions for the reaction in the conventional method involve 120 min. in ethanol in the presence of TEA as a base (Fig 2).

**Table 1:** Optimization of model reaction in the conventional method.

Entry	Base	Solvent	Time (min)	Yield^a^ (%)
1	KOH	MeOH	120	61
2	NaOH	MeOH	120	58
3	K_2_CO_3_	MeOH	120	64
4	NaHCO_3_	MeOH	120	56
5	Et_3_N	MeOH	120	77
6	K_2_CO_3_	EtOH	120	66
**7**	**Et**_**3**_ **N**	**EtOH**	**120**	**79**
8	Et_3_N	EtOH	90	76
9	Et_3_N	EtOH	150	70
10	Et_3_N	EtOH	60	72
11	Et_3_N	CH_2_Cl_2_	120	36
12	Et_3_N	CH_3_CN	120	51
13	Et_3_N	Toluene	120	42

It was hypothesized that enhanced outcomes might be attained by optimizing reaction time and temperature through the utilization of microwave irradiation. This assertion is founded on extant knowledge and prior studies in this field. To this end, a series of trials were conducted on the basis of the aforementioned model reaction. Initially, the reaction temperature was optimized at 75 °C, 100°C and 150 °C for 5 minutes, with partial high reaction yields obtained from all trials. The highest yield, 81%, was obtained at 100 °C (Table 2, Entry 1–3). In this trial, a superior outcome was achieved in comparison to the conventional method. However, experiments were conducted to ascertain the effect of reaction time on yield. The optimum result was obtained with a 10-minute reaction time (Table 2, Entry 4).

**Table 2:** Optimization of microwave irradiated method.

Entry	Temp. (^o^C)	Time (min)	Yield (%)
1	75	5	73
2	100	5	81
3	150	5	76
4	**100**	**10**	**89**
5	100	15	85
6	100	20	80
7	100	30	77

**Fig 3:**
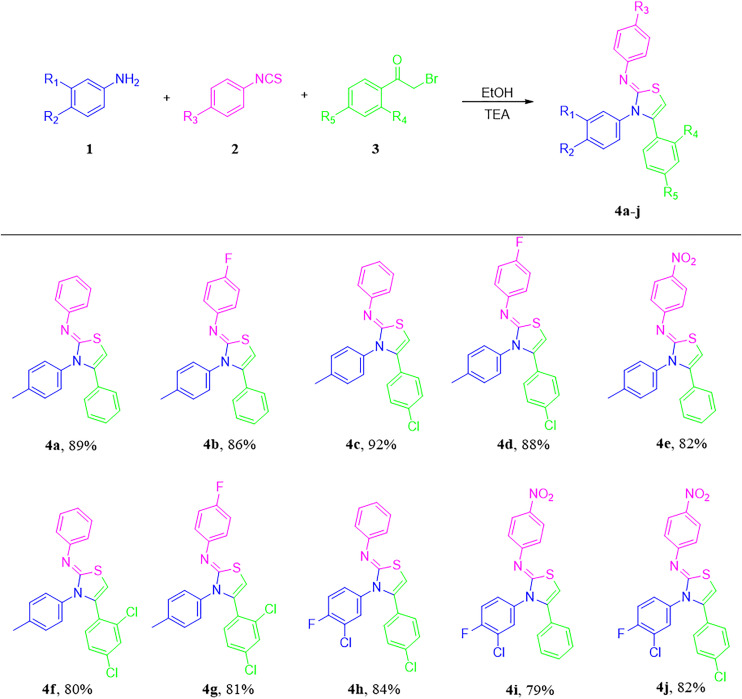
One-pot multi-component microwave-assisted synthesis of thiazole-2-imines.

The optimized conditions were then utilized to synthesize thiazol-2-imine derivatives in remarkable yields (Fig 3).

### 2.2 Green metric calculations

**Fig 4:**
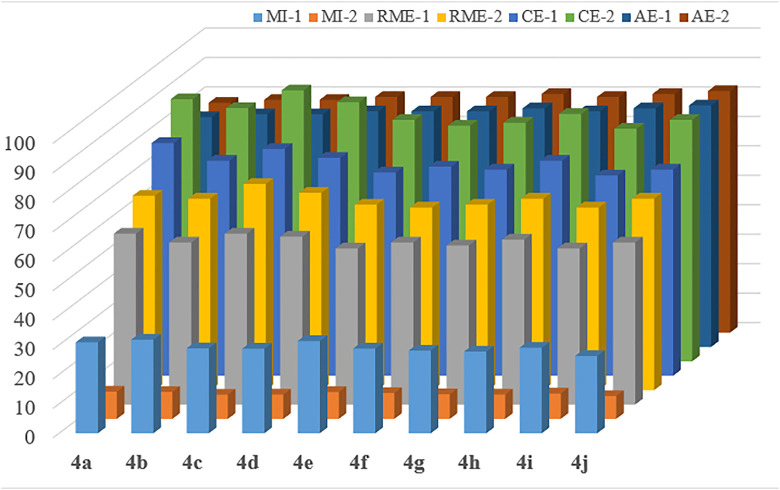
Calculations of Green Metrics (The obtained values are mentioned in the Supp. file).

The definitions of terms related to green chemistry-related, along with metrics by which its success is measured, are regularly updated in contemporary literature [45]. It is widely accepted that metrics should be well-elaborated, fundamental, calculable, and objective, rather than subjective [46–48]. As it is widely acknowledged in the relevant literature, some of the most commonly used metrics include the environmental factor based on molecular weight (Emw), the environmental impact factor based on mass (Em), atom economy (AE), reaction mass efficiency (RME), mass intensity (MI), and carbon efficiency (CE) [48]. The applied reaction protocols (in our study) align with the majority of the green chemistry principles, yielding highly favorable green metrics. The analysis was conducted in accordance with the previously outlined methodology (Fig 4) [49].

The AE% for the microwave irradiation method is superior to that of the conventional method, as indicated by the green metric calculations. This suggests that the microwave irradiation method is the most efficient approach to access the desired products from corresponding raw materials, with minimal waste. In view of the higher reaction yield observed in the microwave method, its CE% value also outperformed that of the conventional route. Analogous outcomes were observed for the RME values. Furthermore, the MI values were found to be elevated for the conventional method, primarily attributable to the greater quantity of solvent utilized in comparison to the microwave method. It is important to emphasize that reactions under microwave conditions can be carried out with significantly less solvent.

### 2.3 Molecular docking and MM-GBSA analysis

EGFR has been identified as a validated and key target for developing anti-cancer agents, as it is generally overexpressed in various several tumour types, including as breast, ovarian, prostate, and colon cancers [50]. Inhibition of EGFR activity has been shown to induce apoptosis in solid tumours, including breast and lung cancers [50–55]. In order to formulate a more potent inhibitor of EGFR, it is crucial to develop a molecular level understanding of kinase-inhibitor interaction [56]. The EGFR TK (tyrosine kinase) domain is structured with an NH_2_-terminal lobe (N-lobe) consisting of 5 β-sheet strands and an Alpha-C-helix consisting residues 729–744. The active site, constituting a fragment of the EGFR, is positioned between residues 855 and 884. In addition, the structure is characterized by the presence of a larger carboxylic acid-terminal lobe (C-lobe), which comprises a total of five α-helices: αE, αF, αG, αH, and αI [57,58]. The ATP-binding site is located between these two lobes, below a well-conserved glycine-enriched phosphate-binding loop that connects β2 and β1in the N-lobe. In the crystal structures, residues Leu718, Val726, Ala743, Met793, and Leu844 exhibit the highest frequency of interactions in the hinge region [59]. Consequently, these elements coalesce to form a hydrophobic and well-conserved core binding pocket. It is also important note that the mutation of the Tyr790 (gatekeeper) residue to methionine is recognized for inducing resistance to inhibitors by enhancing ATP affinity [60].

**Fig 5:**
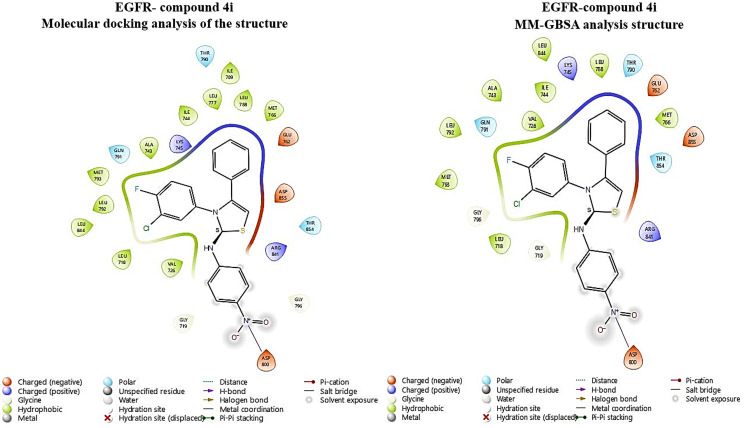
Predicted binding modes and key interactions of 4i in EGFR active site from molecular docking and MM-GBSA analysis.

To elucidate the potential anti-cancer activities of the synthesized compounds and their interaction mechanism with the target protein EGFR at the molecular level, molecular docking was performed. The docking analysis predicted that all compounds interacted with the TK domain (Leu718, Val726, Lys745, Thr790, Gly796, Leu844, Thr854), which has a vital role in the activation mechanism of the target enzyme (Fig S1 in S1 File and Table 3). In particular, compound **4i** demonstrated potent inhibitory effects against EGFR enzymes (binding energy: −6.434) (Table 3). The compound **4i** displayed hydrophobic interaction with Leu844, Ala743, Ile744, Val726, Leu792, Met798, Leu718, Leu788, and Met766 in the EGFR binding site. This compound also exhibited polar interaction with the gatekeeper residue Tyr790. The MM-GBSA results provided strong evidence that the interactions between compound **4i** and the key amino acids (located within the catalytic site of the protein) maintained stability throughout the simulation. This stability ensures the reliability of the ligand within the binding regions of EGFR. Consequently, the interactions crucial for enzyme-ligand binding were maintained within this compound **4i** and EGFR complex structure, further highlighting their significance (Fig 5).

**Table 3:** Interaction residues of EGFR from Molecular docking and MM‐GBSA analysis with docking score and MM-GBSA ΔG (Kcal/mol) scores.

Compound	Docking score	Interaction Residues of EGFR from Molecular Docking Analysis	ΔG score (Kcal/mol)	Interaction Residues of EGFR from MM-GBSAanalysis
4a	−5.868	Leu718, Val726, Ala743, Lys745, Glu762, Met766 Leu788, Thr790, Gln791, Met793, Gly796, Arg841, Asn842, Leu844, Thr854, Asp855, Phe856	−45,57	Val726, Leu844, Met793, Gln791, Ala743, Thr790, Lys745, Leu788, Met766, Glu762, Asp855, Thr854, Phe856, Asn842, Arg841, Gly796, Leu718
4b	−4.998	Leu718, Arg841, Lys745, Ala743, Thr790, Met766, Glu762, Leu788, Asp855, Thr854, Phe856, Val726, Gly796, Asp800, Asn842, Leu844	−44,51	Lys745, Thr790, Glu762, Leu777, Met766, Leu788, Asp855, Thr854, Val726, Ala743, Met793, Gly796, Asp800, Leu844, Leu718, Asn842, Arg841
4c	−5.830	Leu718, Gly719, Leu792, Gly796, Met793, Leu844, Ala743, Thr790, Thr854, Glu762, Asp855, Lys745, Val726, Pro794	−45,73	Leu718, Gly796, Leu792, Met793, Gln791, Leu844, Ala743, Thr790, Asp855, Lys745, Glu762, Val726, Thr854,Gly719, Pro794
4d	−5.924	Leu718, Gly719, Pro794, Gly796, Met793, Leu792, Ala743, Leu844, Thr790, Asp855, Lys745, Thr854, Glu762, Val726	−45,06	Leu718, Gly796, Leu792, Met793, Ala743, Gln791, Leu844, Thr790, Asp855, Glu762, Lys745, Thr854, Val726, Gly719, Pro794
4e	−6.056	Leu718, Gly719, Pro794, Phe795, Gly796, Met793, Leu792, Val726, Ala743, Leu788, Thr790, Glu762, Lys745, Met766, Thr854, Asp855, Leu844, Arg841, Asp800	−48,39	Pro794, Gly796, Leu792, Met793, Val726, Ala743, Thr790, Leu788, Glu762, Lys745, Met766, Asp855, Thr854, Leu844, Asp800, Arg841, Leu718
4f	−5.198	Val843, Gly796, Leu718, Met793, Ala743, Gln791, Leu792, Thr790, Met766, Val726, Lys745, Thr854, Asp855, Leu844, Asp842, Arg841, Leu798, Leu799, Asp800, Ala840	−33,13	Leu844, Lys745, Gly796, Leu718, Ala743, Met793, Thr854, Thr790, Met766, Val726, Asp855, Asn842, Arg841, Leu798, Ala840, Leu799, Asp800, Val843
4g	−5.216	Gly796, Leu718, Met793, Leu844, Ala743, Thr790, Thr854, Met766, Val726, Lys745, Asp855, Arg841, Asn842, Leu798, Val843, Asp800, Leu799, Ala840	−35,28	Leu844, Gly796, Leu718, Met793, Ala743, Thr790, Thr854, Met766, Val726, Asn842, Lys745, Asp855, Arg841, Leu798, Val843, Ala840, Leu799, Asp800
4h	−5.402	Gly724, Thr725, Lys745, Ala743, Val726, Thr790, Leu788, Met766, Glu762, Phe856, Thr854, Asp855, Leu844, Leu718, Gly796, Arg841, Asn842	−39,86	Gly724, Thr725, Lys745, Val726, Ile744, Ala743, Thr790, Met766, Leu788, Asp855, Thr854, Glu762, Leu844, Met793, Leu718, Gly796, Arg841, Asn842
4i	−6.434	Leu718, Gly719, Gly796, Met793, Leu792, Gln791, Ala743, Val726, Ile744, Leu844, Lys745, Leu788, Thr790, Glu762, Met766, Thr854, Asp855, Arg841, Asp800	−53,40	Val726, Leu718, Leu844, Leu792, Met793, Gln791, Ala743, Lys745, Lys745, Ile744, Leu777, Ile789, Thr790, Leu788, Met766, Glu762, Asp855, Thr854, Arg841, Asp800, Gly796
4j	−6.167	Leu718, Gly719, Val726, Pro794, Gly796, Met793, Leu792, Leu844, Ala743, Thr790, Thr854, Lys745, Glu762, Asp855	−48,18	Pro794, Leu718, Gly796, Met793, Leu792, Leu844, Ala743, Gln791, Lys745, Thr790, Glu762, Asp855, Thr854, Gly719, Val726

In addition, the free binding energy was obtained from MM-GBSA calculations of the poses derived from docking, which considers the effects of implicit solvents and provides a more accurate estimation of ligand-receptor affinity. The MM-GBSA free binding energy for compound **4i** and EGFR was calculated to be the highest among the compounds tested, with an ΔG score of −53.40 kcal/mol, a finding that is analogous to the docking scores.

### 2.4 Molecular dynamics simulation

**Fig 6:**
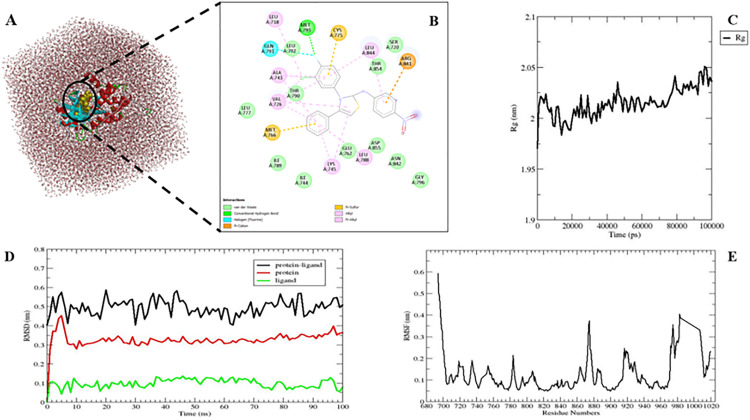
(A) 3D structure of EGFR–4i in solvation box. (B) 2D interactions of MD conformations of EGFR–4i. (C) *R*g analysis of complex structure during the simulation duration (100 ns). (D) The RMSD trajectory of complex, protein and compound structures. Black color represents protein and ligand (complex), red color signifies protein and green color denotes ligand (compound 4i) structures. (E) The RMSF profile of EGFR in complex structures.

To assess the dynamic behavior and binding stability of the EGFR–compound **4i** complex, molecular dynamics (MD) simulations were performed. Compound **4i**, identified as the ligand with the most favorable binding energy, was selected for this analysis to gain detailed insights into its interactions with the receptor’s active site. Intermolecular forces are critical in stabilizing ligand conformations within protein binding pockets. Throughout the 100 ns molecular dynamics simulation, electrostatic and Van der Waals interactions with residues Ile744, Glu762, Thr790, Leu792, Thr854, and Asp855, as well as π-alkyl interactions with Leu718, Val726, Ala743, Lys745, Leu788, and Leu844, were stably maintained. Notably, a stable and strong hydrogen bond interaction with Met793 play a significant role towards complex’s stability. This analysis suggests that compound **4i** forms stable interactions with key amino acid residues involved in the activation mechanism of the target enzyme. In addition, a root mean square fluctuation (RMSF) analysis was conducted for each residue of the EGFR protein in complex with compound **4i**. RMSF is a quantitative measure used to assess the thermal mobility of individual residues relative to their reference positions during the simulation. The analysis revealed that the catalytically significant residues of EGFR, namely Leu718, Val726, Ala743, Met793, and Leu844 in the hinge region, exhibited minimal fluctuations (Fig 6E). These findings indicate that the binding of compound 4i did not result in substantial conformational shifts in the active site residues of EGFR, thereby confirming that the protein-ligand complex sustained the structural stability (throughout the simulation period). To monitor conformational changes, the structural stability of the system was assessed via root mean square deviation (RMSD) analysis of the backbone Cα atoms. The RMSD of the EGFR–**4i** complex fluctuated between 0.4 and 0.6 Å, while individual protein and ligand RMSD values remained within 0.1–0.5 Å (Fig 6D), indicating no significant deviations occurred during the simulation. These results suggest that compound **4i** maintained a stable conformation while remaining consistently bound to the active site. The radius of gyration (Rg) was also computed to analyze the overall compactness of the protein structure. The Rg values of EGFR fluctuated between 1.97 and 2.05 nm throughout the simulation, indicating that the protein retained a stable and compact form over time (Fig 6C).

These findings demonstrate that compound **4i** forms stable interactions with key residues in the catalytic domain of EGFR, maintaining its binding integrity throughout the simulation. These results support its potential as a reliable and effective EGFR inhibitor candidate in targeted drug design efforts.

### 2.5 In silico toxicity prediction

The toxicity prediction analysis was carried out using the web-based online platform ProTox-3.0 (https://tox.charite.de/protox3/) and pkCSM [61]. The toxicity calculations of compound 4i, which was found to have the best anti-cancer activity, predicted toxicity results of 6.

The hERG1 potassium channel is critical in regulating cardiac electrical activity, and its inhibition may result in severe cardiac arrhythmias. Therefore, interaction with the hERG1 channel constitutes a key safety parameter that must be evaluated during the drug development [61]. In the present analysis, none of the tested compounds were predicted to significantly inhibit the hERG1 channel, suggesting a favorable cardiotoxicity profile. On the other hand, hepatotoxicity assessment serves as a valuable tool for predicting the potential of a compound to impair normal hepatic physiological functions. Among the evaluated compounds, **4c**, **4e**, **4f**, **4h**, and **4j** were identified as non-hepatotoxic (Fig S1b in S1 File, S1 Table in S1 File). (Fig S1b in S1 File, S1 Table in S1 File).

### 2.6 Anti-cancer activity

**Fig 7:**
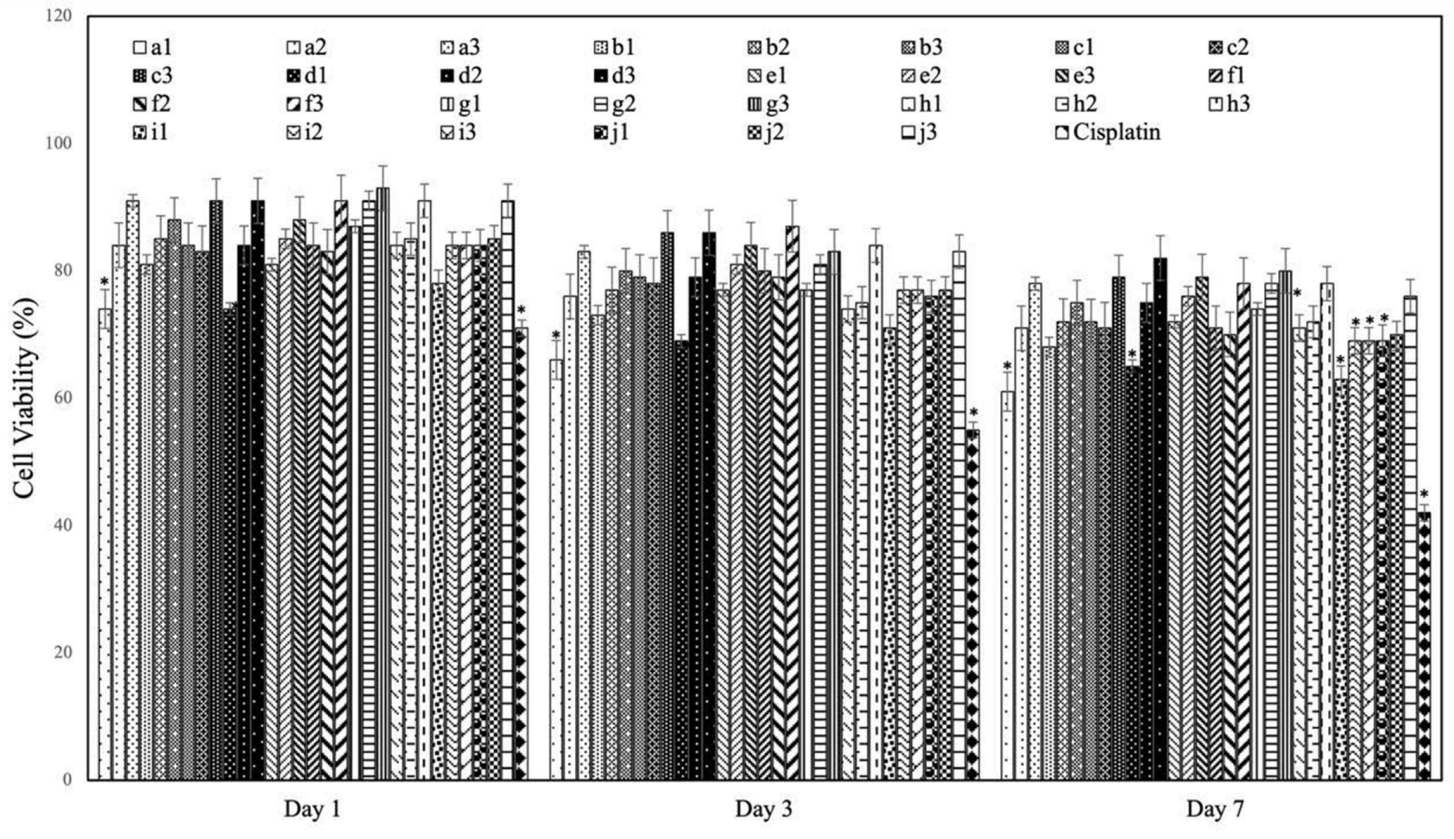
Saos-2 cells viability relative to cells grown on positive control TCPS (*n* = 4). * the samples which shows statically significant difference with positive control.

Cancer cell lines are of significant importance as commonly utilized models in cancer research and the development of medications. They remain crucial in contemporary cancer studies and serve as vital preclinical model systems for enhancing our understanding of mechanistic and therapeutic processes. The objective of this study was to assess the cytotoxic impact of our recently synthesized compounds on the SaOs-2 cell line in an *in vitro* setting. As demonstrated by the cell viability assay, relative cell viability was decreased by increasing concentration of all synthesized compounds in which 1000 µg/ml concentration is most effective dosage for each group. The figure indicates a marked decline in relative cell viability with increasing incubation time. Moreover, it was observed that Group A exhibited a substantial decline in cell viability when compared to the other groups. At the conclusion of the seven-day period, the cell viability of Group A was recorded to be approximately 60%. It can thus be concluded that anti-cancer effect of synthesized thiazole derivatives depends on concentration and time of incubation. The results clearly proved that increasing incubation time significantly decrease SaOs-2 cells viability (Fig 7).

According to a study by Moghadam-Manesh et al., the survival of breast cancer cells is influenced by the concentration of heterocycles and the duration of incubation. Specifically, high concentrations of thiazole derivatives can decrease both the proliferation and viability of the cells [62,63]. These compounds exhibit various mechanisms of action, such as inhibiting cell proliferation, inducing apoptosis, and interfering with cancer cell signalling pathways. Thiazole derivatives have been determined as a potentially promising class of compounds to develop new anti-cancer agents as they have been found to affect the cancer cells selectively (sparing normal cells). Previous studies proved that thiazole derivatives have a potential to be used as anti-fungal, anti-microbial, anti-oxidant, anti-tubercular and anti-cancer agents. Also, it is known that thiazole derivatives (1,3-thiazole and 1,3,4-thiazole) exhibit considerable anti-tumor activity. Gomha et al. was revealed that thiazole derivatives which has bromine in their structure show less cytotoxicity effect in comparison with compounds with chlorine contents [64]. So, it can be concluded that presence of chlorine in structure has considerable effect on anti-cancer activity. It was concluded that the migration and occupation of cancer cells were hindered by thiazole derivatives through their ability to disrupt the movement of the cell’s cytoskeleton, which resulted in a decrease in the simultaneous presence of the actin-bundling protein fascin [65].

Wang and co-workers investigated that *N*-phenyl-4-(thiazol-5-yl)pyrimidin-2-amines exhibited anti-cancer effect via enhanced polyploidy and mitotic failure [66]. Thiazole containing compounds such as 2-(3-Indolyl)- *N*-arylthiazole-4-carboxamides, (*E*)-4-(2,4-dichlorophenyl)-2-(2-(4-(trifluoromethyl) benzylidene)hydrazinyl)thiazole, 2-(2-(pyridin-2-ylmethylene)hydrazinyl)-4-(4-chloro phenyl)-1,3-thiazole, and (2-(1-(pyridin- 2-yl)ethylene)hydrazinyl)-4-(4-chlorophenyl)-1,3-thiazole have been notified as anti-tumour and apoptosis agents [67]. All these studies clearly demonstrate the accuracy of the results of the in vitro research in our study. The cell viability also proved that (*Z*)-4-fluoro-*N*-(4-phenyl-3-(p-tolyl)thiazol-2(3*H*)-ylidene)aniline is a thiazole derivative with potential anti-cancer properties. The presence of a fluoro substituent often enhances the biological activity and selectivity of such compounds. Cell viability results show a significant reduction in cell number after a 7-day incubation period.

The observed decrease in the number of SaOs-2 cells following incubation with *N*-(4-(4-chlorophenyl)-3-(p-tolyl)thiazol-2(3H)-ylidene)aniline can be attributed to the biological effects of the chlorophenyl group on cancer cells. It has been established that the chlorophenyl group exerts a number of biological effects when incorporated into various compounds [68]. Indeed, the group has been shown to enhance the cytotoxic activity of a compound, thereby increasing its effectiveness in the elimination of cancer cells. It also improves the compound’s ability to penetrate cell membranes, ensuring better access to intracellular targets. Furthermore, the chlorophenyl group can disrupt critical signalling pathways in cancer cells, including those associated with cell proliferation, apoptosis, and metastasis, ultimately inhibiting tumor growth and spread [68].

**Fig 8:**
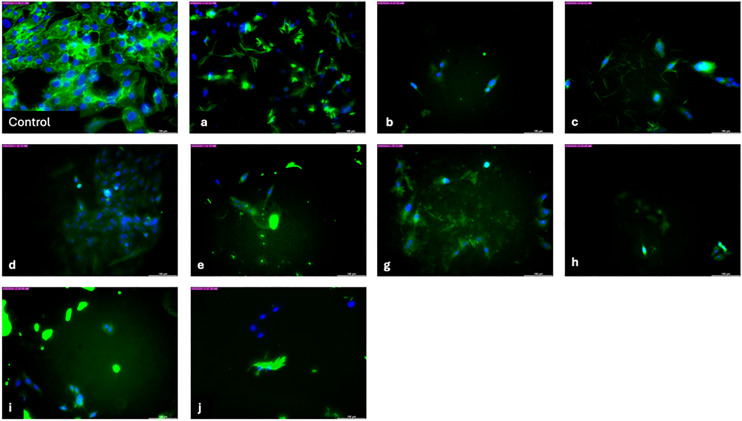
Light microscopy images of SaOs-2 cell after incubation with synthesized compounds (Magnification is 20X and scale bar is 100 μm).

As illustrated, the shape analysis of SaOs-2 cells following incubation with the synthesized compound for a period of three days is demonstrated. The images clearly showed that there is considerable decrease in cells number in comparison with control group. The results proved that all compounds have cytotoxic effect on cancer cell. However, further investigation is required to ascertain the precise biological effect of the compounds. There is no change in morphology of SaOs-2 cell while significant decline was seen for all groups (Fig 8).

It is evident from the IC_50_ results that all compound exhibited comparable anti-cancer activity. However, the compounds **4i**, **4d** and **4b** were interpreted to be most promising, thereby displaying IC_50_ values of 0.190 ± 0.045, 0.212 ± 0.006 and 0.214 ± 0.009 (μg/mL), respectively. In consideration of the structure-activity relationship, the introduction of *p*-fluorophenyl to the 2^nd^ position of thiazole ring resulted in an enhancement of activity (**4b**). Conversely, the presence of p-chlorophenyl led to a reduction in activity (**4c**). The replacement of fluorine with nitro group at the 2^nd^ position of Ph ring, also caused a decrease in activity (**4e**). Furthermore, the incorporation of a disubstituted (chlorine) phenyl group at the 2^nd^ position of the thiazole ring did not improve the activity. It was observed that the substitution of toluidine at third position of thiazole ring with 3-chloro-4-fluoro aniline did not cause significant changes in activity. However, compared to compound **4e**, the nitro group in compound **4i** increased the activity (Table 4).

**Table 4:** IC_50_ results of synthesized compound after incubation for 24 h.

Comp.	IC_50_ (μg/mL)	Comp.	IC_50_ (μg/mL)
**4a**	0.244 ± 0.012	**4f**	0.264 ± 0.035
**4b**	0.214 ± 0.009	**4g**	0.240 ± 0.005
**4c**	0.240 ± 0.028	**4h**	0.232 ± 0.012
**4d**	0.212 ± 0.006	**4i**	0.190 ± 0.045
**4e**	0.258 ± 0.022	**4j**	0.273 ± 0.029
**Paclitaxel**	0.170 ± 0.002		

## 3. Conclusion

In this study, we have synthesized novel thiazole-2-imine derivatives via microwave irradiation method in comparison with conventional method. The findings indicated a substantial decrease in reaction time, with a magnitude of 12-fold, concomitant with an augmentation in yield of approximately 15%. Anti-cancer activity studies of the synthesized derivatives were evaluated against Saos-2 cell line, and all compounds exhibited promising results. Among them, the compound **4i** was found to have highest inhibitory potential with an IC_50_ value of 0.190 ± 0.045 μg/mL. According to the morphology examination, a significant decrease in cell number was observed in all of the compound groups, in comparison with the control group. Furthermore, it was indicated that the compound **4i** was found to illustrate the strongest binding affinity towards the target protein in molecular docking and MM-GBSA analyses. These encouraging results suggested that further studies should be conducted to modify these compounds with a view to generate more effective anti-cancer agents.

## 4. Experimental

### 4.1 General synthetic protocol for the preparation of compounds 4(a-j)

**Method 1.** To the reaction mixture of p-toluidine or 3-chloro-4-fluoro aniline (1 mmol) and corresponding aryl isothiocyanate (1 mmol) in ethanol (5 ml), phenacyl bromide (1 mmol) and Et_3_N (0.5 mmol) were inserted appropriately. The resulting mixture was subjected to reflux. Once the reaction had reached completion, the progress was monitored by thin-layer chromatography (TLC). Reaction mixture was then cooled and resulting precipitates were filtered and dried, thus producing the respective thiazole-2-imine derivatives without the necessity for further purification.

**Method 2.** The mixture of *p*-toluidine or 3-chloro-4-fluoro aniline (1 mmol) and corresponding aryl isothiocyanate (1 mmol) in 3 ml of ethanol was firstly irradiated at 100 °C for 2 min in monomode microwave reactor. Then, appropriate phenacyl bromide (1 mmol) and Et_3_N (0.2 mmol) were introduced to the mixture, which was irradiated for 8 more minutes at the same conditions. Once the reaction had reached completion (monitored by TLC), reaction mixture was cooled. Upon cooling, the resulting precipitates were filtered and dried, thus producing the respective thiazole-2-imine derivatives without the necessity for further purification.

#### 4.1.1 *N*-(4-phenyl-3-(*p*-tolyl)thiazol-2(3*H*)-ylidene)aniline (4a).

Yield (MW): 89%, Yield (CM): 79%. FT-IR (υ_max_, cm^-1^): 3073, 2919, 1580. ^1^H NMR (DMSO-*d*_6_, δppm): 2.26 (s, 3H, CH_3_), 6.45 (s, 1H, CH), 6.82 (d, 2H, **J* *= 8.0 Hz, ArH), 7.12 (d, 2H, **J* *= 8.0 Hz, ArH), 7.16 (s, 2H, ArH), 7.23-7.29 (m, 4H, ArH), 7.35 (d, 2H, **J* *= 8.0 Hz, ArH), 7.38 (s, 2H, ArH). ^13^C NMR (DMSO-*d*_6_, δppm): 20.94 (CH_3_), 99.85 (CH), ArC: [121.38 (2CH), 128.13 (CH), 128.66 (d, **J* *= 6.0 Hz, CH), 128.85 (CH), 129.22 (CH), 129.30 (2CH), 129.51 (2CH), 129.83 (CH), 129.99 (CH), 130.49 (2CH), 131.56 (C), 132.44 (C), 138.27 (C), 139.69 (C)], 151.13 (thiazole C-4), 160.97 (thiazole C-2). EI MS *m/z* (%): 343 ([M + 1]^+^, 100%).

#### 4.1.2 4-fluoro-*N*-(4-phenyl-3-(*p*-tolyl)thiazol-2(3*H*)-ylidene)aniline (4b).

Yield (MW): 86%, Yield (CM): 73%. FT-IR (υ_max_, cm^-1^): 3107, 2922, 1581. ^1^H NMR (DMSO-*d*_6_, δppm): 2.28 (s, 3H, CH_3_), 6.46 (s, 1H, CH), 6.92-6.95 (m, 1H, ArH), 7.11-7.22 (m, 8H, ArH), 7.25-7.27 (m, 4H, ArH). ^13^C NMR (DMSO-*d*_6_, δppm): 21.10 (CH_3_), 98.05 (CH), ArC: [116.19 (d, **J* *= 23.0 Hz, CH), 116.57 (d, **J* *= 22.0 Hz, CH), 121.07 (d, **J* *= 8.0 Hz, CH), 128.70 (d, **J* *= 3.0 Hz, 2CH), 128.76 (CH), 128.88 (CH), 129.20 (2CH), 129.83 (2CH), 130.49 (CH), 131.56 (C), 131.67 (d, *J* = 9.0 Hz, C), 135.61 (C), 137.54 (C), 139.88 (C), 157.48-159.92 (d_C-F_ = 244.0 Hz, C)], 153.83 (thiazole C-4), 162.63 (thiazole C-2). EI MS *m/z* (%): 361 ([M]^+^, 100%).

#### 4.1.3 *N*-(4-(4-chlorophenyl)-3-(*p*-tolyl)thiazol-2(3*H*)-ylidene)aniline (4c).

Yield (MW): 92%, Yield (CM): 77%. FT-IR (υ_max_, cm^-1^): 3077, 2922, 1584. ^1^H NMR (DMSO-*d*_6_, δppm): 2.29 (s, 3H, CH_3_), 6.51 (s, 1H, CH), 6.82 (d, 1H, **J* *= 8.0 Hz, ArH), 6.92 (d, 1H, *J* = 8.0 Hz, ArH), 7.03 (t, 1H, *J* = 12.0 Hz, ArH), 7.11-7.20 (m, 5H, ArH), 7.30-7.40 (m, 5H, ArH). ^13^C NMR (DMSO-*d*_6_, δppm): 21.13 (CH_3_), 99.92 (CH), ArC: [121.38 (CH), 121.51 (2CH), 123.56 (CH), 128.79 (2CH), 129.19 (2CH), 129.45 (d, **J* *= 8.0 Hz, CH), 129.98 (d, **J* *= 7.0 Hz, CH), 130.43 (2CH), 130.50 (CH), 133.58 (C), 135.46 (C), 137.68 (C), 138.55 (C), 144.04 (C)], 151.29 (thiazole C-4), 160.00 (thiazole C-2). EI MS *m/z* (%): 102 (100%), 377 ([M + 1]^+^, 32%).

#### 4.1.4 (*Z*)-*N*-(4-(4-chlorophenyl)-3-(*p*-tolyl)thiazol-2(3*H*)-ylidene)-4-fluoroaniline (4d).

Yield (MW): 88%, Yield (CM): 74%. FT-IR (υ_max_, cm^-1^): 3077, 2995, 1587. ^1^H NMR (DMSO-*d*_6_, δppm): 2.30 (s, 3H, CH_3_), 6.71 (s, 1H, CH), 7.05 (dd, 2H, **J* *= 32.0 Hz, ArH), 7.18-7.24 (m, 8H, ArH), 7.32-7.38 (m, 2H, ArH). ^13^C NMR (DMSO-*d*_6_, δppm): 21.12 (CH_3_), 108.91 (CH), ArC: [114.82 (2CH), 122.41 (2CH), 128.26 (2CH), 129.21 (2CH), 129.51 (2CH), 129.57 (2CH), 133.80 (C), 134.66 (C), 138.49 (C), 138.55 (C), 137.22 (C), 157.68 and 159.73 (d_C-F,_ *J* = 205.0 Hz, C)], 150.41 (thiazole C-4), 157.08 (thiazole C-2). EI MS *m/z* (%): 102 (95%), 395 (100%), 397 ([M]^+^, 45%).

#### 4.1.5 4-nitro-*N*-(4-phenyl-3-(*p*-tolyl)thiazol-2(3*H*)-ylidene)aniline (4e).

Yield (MW): 82%, Yield (CM): 69%. FT-IR (υ_max_, cm^-1^): 3104, 2917, 1508. ^1^H NMR (DMSO-*d*_6_, δppm): 2.28 (s, 3H, CH_3_), 6.71 (s, 1H, CH), 7.21 (d, 6H, **J* *= 4.0 Hz, ArH), 7.27 (d, 5H, **J* *= 4.0 Hz, ArH), 8.19 (d, 2H, **J* *= 8.0 Hz, ArH). ^13^C NMR (DMSO-*d*_6_, δppm): 21.13 (CH_3_), 99.33 (CH), ArC: [122.17 (2CH), 126.02 (2CH), 128.76 (2CH), 128.97 (2CH), 129.47 (2CH), 129.99 (2CH), 130.61 (CH), 131.08 (C), 135.10 (C), 138.16 (C), 140.17 (C), 142.46 (C)], 157.13 (thiazole C-4), 161.81 (thiazole C-2). EI MS *m/z* (%): 106 (89%), 388 ([M + 1]^+^, 100%), 389 ([M + 2]^+^, 79%).

#### 4.1.6 *N*-(4-(2,4-dichlorophenyl)-3-(*p*-tolyl)thiazol-2(3*H*)-ylidene)aniline (4f).

Yield (MW): 80%, Yield (CM): 71%. FT-IR (υ_max_, cm^-1^): 3081, 2921, 1585. ^1^H NMR (DMSO-*d*_6_, δppm): 2.24 (s, 3H, CH_3_), 6.48 (s, 1H, CH), 6.93 (d, 2H, **J* *= 8.0 Hz, ArH), 7.03 (t, 1H, **J* *= 12.0 Hz, ArH), 7.12 (d, 2H, **J* *= 8.0 Hz, ArH), 7.18 (d, 2H, **J* *= 8.0 Hz, ArH), 7.32 (t, 2H, **J* *= 16.0 Hz, ArH), 7.42 (d, 1H, *J* = 8.0 Hz, ArH), 7.53 (s, 1H, ArH), 7.57 (d, 1H, **J* *= 8.0 Hz, ArH). ^13^C NMR (DMSO-*d*_6_, δppm): 21.08 (CH_3_), 99.80 (CH), ArC: [121.47 (2CH), 123.53 (CH), 127.77 (CH), 128.98 (2CH), 129.34 (CH), 129.65 (2CH), 129.89 (C), 130.00 (2CH), 134.57 (CH), 134.71 (d, *J* = 3.0 Hz, 2C), 135.23 (C), 135.32 (C), 137.80 (C)], 151.41 (thiazole C-4), 159.19 (thiazole C-2). EI MS *m/z* (%): 106 (71%), 149 (48%), 157 (100%), 213 (75%), 411 ([M]^+^, 48%), 413 ([M + 2]^+^, 32%).

#### 4.1.7 *N*-(4-(2,4-dichlorophenyl)-3-(*p*-tolyl)thiazol-2(3*H*)-ylidene)-4-fluoroaniline (4g).

Yield (MW): 81%, Yield (CM): 70%. FT-IR (υ_max_, cm^-1^): 3091, 2958, 1589. ^1^H NMR (DMSO-*d*_6_, δppm): 2.25 (s, 3H, CH_3_), 6.53 (s, 1H, CH), 6.97 (t, 2H, **J* *= 12.0 Hz, ArH), 7.17-7.20 (m, 6H, ArH), 7.42 (d, 1H, **J* *= 8.0 Hz, ArH), 7.54 (d, 1H, **J* *= 8.0 Hz, ArH), 7.59 (s, 1H, ArH). ^13^C NMR (DMSO-*d*_6_, δppm): 21.08 (CH_3_), 100.51 (CH), ArC: [116.51 (CH), 116.73 (CH), 123.17 (d, **J* *= 7.0 Hz, CH), 127.78 (CH), 128.94 (2CH), 129.34 (CH), 129.70 (2CH), 130.54 (CH), 134.49 (C), 134.59 (C), 134.73 (C), 135.30 (C), 135.47 (C), 137.96 (C), 141.95 (C)], 157.99 (thiazole C-4), 159.97 (thiazole C-2). EI MS *m/z* (%): 102 (70%), 429 ([M]^+^, 100%), 431 ([M + 2]^+^, 71%).

#### 4.1.8 *N*-(3-(3-chloro-4-fluorophenyl)-4-(4-chlorophenyl)thiazol-2(3*H*)-ylidene)aniline (4h).

Yield (MW): 84%, Yield (CM): 73%. FT-IR (υ_max_, cm^-1^): 3086, 2986, 1603. ^1^H NMR (DMSO-*d*_6_, δppm): 6.59 (s, 1H, CH), 6.92 (bs, 1H, ArH), 7.10 (d, 1H, **J* *= 8.0 Hz, ArH), 7.16 (d, 2H, **J* *= 4.0 Hz, ArH), 7.28 (s, 1H, ArH), 7.32 (d, 5H, **J* *= 8.0 Hz, ArH), 7.38 (d, 2H, **J* *= 8.0 Hz, ArH). ^13^C NMR (DMSO-*d*_6_, δppm): 99.18 (CH), ArC: [118.00 (CH), 118.21 (CH), 120.35 (C), 121.92 (d, **J* *= 7.0 Hz, CH), 123.14 (CH), 128.41 (2CH), 128.80 (2CH), 129.45 (d, *J* = 3.0 Hz, 2CH), 130.21 (C), 130.48 (2CH), 133.71 (C), 137.81 (C), 138.59 (C), 152.50-154.90 (d_C-F_, *J* = 240.0 Hz, C)], 149.05 (thiazole C-4), 161.17 (thiazole C-2). EI MS *m/z* (%): 416 ([M]^+^, 100%).

#### 4.1.9 *N*-(3-(3-chloro-4-fluorophenyl)-4-phenylthiazol-2(3*H*)-ylidene)-4-nitroaniline (4i).

Yield (MW): 79%, Yield (CM): 68%. FT-IR (υ_max_, cm^-1^): 3108, 1541. ^1^H NMR (DMSO-*d*_6_, δppm): 6.68 (s, 1H, CH), 7.18 (d, 2H, *J* = 8.0 Hz, ArH), 7.22 (d, 2H, **J* *= 4.0 Hz, ArH), 7.32 (s, 4H, ArH), 7.44 (t, 1H, **J* *= 16.0 Hz, ArH), 7.74 (d, 1H, **J* *= 4.0 Hz, ArH), 8.20 (d, 2H, **J* *= 8.0 Hz, ArH). ^13^C NMR (DMSO-*d*_6_, δppm): 99.35 (CH), ArC: [117.50 (d, d, **J* *= 22.0 Hz, CH), 120.01 (d, **J* *= 19.0 Hz, C), 122.15 (2CH), 126.04 (2CH), 128.90 (2CH), 129.11 (2CH), 129.36 (CH), 130.57 (d, *J* = 8.0 Hz, CH), 130.76 (C), 131.98 (CH), 134.76 (d, **J* *= 3.0 Hz, C), 139.46 (C), 142.55 (C), 155.74-158.20 (d_C-F_, **J* *= 246.0 Hz, C)], 157.39 (thiazole C-4), 161.27 (thiazole C-2). EI MS *m/z* (%): 427 ([M + 1]^+^, 100%).

#### 4.1.10 *N*-(3-(3-chloro-4-fluorophenyl)-4-(4-chlorophenyl)thiazol-2(3*H*)-ylidene)-4-nitro aniline (4j).

Yield (MW): 82%, Yield (CM): 70%. FT-IR (υ_max_, cm^-1^): 3110, 1576. ^1^H NMR (DMSO-*d*_6_, δppm): 6.74 (s, 1H, CH), 7.18 (d, 2H, **J* *= 8.0 Hz, ArH), 7.24 (d, 2H, **J* *= 8.0 Hz, ArH), 7.36 (m, 1H, ArH), 7.41 (d, 2H, **J* *= 8.0 Hz, ArH), 7.45 (t, 1H, **J* *= 16.0 Hz, ArH), 7.79 (d, 1H, **J* *= 8.0 Hz, ArH), 8.20 (d, 2H, **J* *= 8.0 Hz, ArH). ^13^C NMR (DMSO-*d*_6_, δppm): 100.30 (CH), ArC: [117.60 (d, **J* *= 22.0 Hz, CH), 120.17 (d, **J* *= 19.0 Hz, C), 122.22 (2CH), 126.05 (2CH), 129.00 (2CH), 129.53 (C), 130.53 (d, **J* *= 8.0 Hz, CH), 130.91 (2CH), 132.00 (CH), 134.18 (C), 134.43 (d, **J* *= 3.0 Hz, C), 138.30 (C), 142.72 (C), 155.86-158.33 (d_C-F_, **J* *= 247.0 Hz, C)], 156.94 (thiazole C-4), 161.37 (thiazole C-2). EI MS *m/z* (%): 461 ([M]^+^, 100%).

### 4.2 Molecular modeling

To elucidate detailed molecular interactions that underpin the enzyme-ligand binding mechanism, molecular docking is employed as a structural framework-based drug design approach in the context of molecular biology and drug discovery. It is perative to gain an understanding of the 3D structures of the target enzyme and ligand. Consequently, the three-dimensional crystal structures of the epidermal growth factor receptor (EGFR) target enzyme complex with gefitinib were obtained from the PDB (Protein Data Bank) website (http://www.rcsb.org/pdb) (PDB ID: 4WKQ and resolution: 1.85 Å) and Protein Preparation Wizard of Maestro, version 13.1 (2022−4; Schrödinger LLC) was used for its preparation [69]. Using the binding position of the ligand (gefitinib) in the crystal complex structure, the active site of the protein structure was determined. Subsequently, the receptor grid generation module of Maestro was utilized to generate active site grids for the protein structure, with the central coordinates set as 2.436, 193.199, and 21.770. Ten new thiazole derivative compounds (**4a-4j**) were subjected to optimization using the Ligprep module of the Maestro software, version 13.1 [70], the module for geometric refining of ligands is designed to build 3D structures with correct chirality. Epic Software [71] was used at pH 7.0 + /- 2.0 to formulate possible ionization states of ligands, tautomers, stereoisomers, and conformers. The OPLS4 [72] force field was used for the restrained minimization of the resulting conformers. Molecular docking was conducted using Glide [73] at standard precision (SP) after the completion of enzyme and synthesized compound structure preparations. The MM-GBSA method using Prime [74] software and the VSGB solvation model with OPLS4 force field were utilized to calculate the free binding energy for the best poses obtained from docking. In this analysis, the docking score, and the MM-GBSA binding free energy (ΔG) were estimated. To evaluate the dynamic behavior and binding stability of the EGFR–compound **4i** complex, **GROMACS 5.0.7** was employed to perform MD simulations [75]. Ligand topology files were generated using the CGenFF program [76], the protein topology was constructed with the CHARMM36 force field [77], and the system was solvated using the TIP3P water model (Fig 6A). The system was placed in a dodecahedral box under periodic boundary conditions, with Na⁺ ions added to neutralize the total charge. The simulation was performed in three stages: energy minimization, equilibration, and production. Energy minimization step was performed using the steepest descent algorithm for 1000 steps to eliminate steric clashes in the system. During the equilibration phase, the system was subjected to two 100 ps simulations under NVT (constant volume-temperature) and NPT (constant pressure-temperature) conditions. The production simulation, conducted after equilibration, lasted 100 ns with a 2-fs time step, during which the structural stability and flexibility of the system were assessed through RMSD, RMSF, and Rg analyses.

### 4.3 Anti-cancer activity

Cell viability was ascertained using the osteosarcoma cell line (SaOs2- HTB-85™, ATCC, USA). The cells were cultured in DMEM supported with 10% FBS and 1% penicillin/streptomycin, and presented in a controlled humid environment containing 5% CO_2_ at 37 °C. For the in vitro analysis, the cells were washed with phosphate-buffered saline (PBS), harvested through trypsinization, and seeded into 96-well plates at 1000 cells per well density. They were then incubated under 5% CO_2_ and 95% air at 37 °C for 24 hours.

The stock solution **(10 mg/mL)** for each sample was prepared in dimethylsulphoxide (DMSO). Cells were treated with serial dilutions were made from stock solution by cell culture medium to give working concentrations of **1000**, **500**, **100** μg/mL (Table 1) and seeded cells were subjected to incubation for one, three and seven days at 5% CO_2_ and 95%. The growth media was renewed every 2 days. At the end of each incubation period, wells were rinsed twice with PBS and were subjected to incubation for 4 h in PrestoBlue solution containing growth media. The absorbance of the samples was determined by using a mQuantTM microplate spectrophotometer from Biotek Instruments Inc, USA. The Saos-2 cells viability on each disc was evaluated (*n* = 4). Additionally, the morphology of Saos-2 cells cultured on plates was analyzed after three days of incubation. The cells were stained with phalloidin-FITC (Abcam, UK) and DAPI (Abcam, UK) and examined using a laser-assisted light microscope (DMI 8, Leica, Netherlands).

### 4.4 Statistical analysis

To ascertain the impact of synthesised compounds on cell viability, an ANOVA test was conducted at a significance level of 0.05 utilising Minitab 17 statistical software (Minitab Ltd., UK) for the statistical analysis (*n* = 4).

## Supporting information

S1 File Fig. S1. Molecular docking results of all compounds. Fig. S2. Toxicity prediction results. Fig. S3 FTIR, MS and NMR spectra of all compounds. Table S1. In silico hepatoxicity prediction results. Table S2 and Table S3. Green metric calculations.
(DOCX)
