# Supplementary material for: Novel thiazole derivatives as effective anti-cancer agents against human osteosarcoma cell line (SaOS-2): Microwave-assisted one-pot three-component synthesis, in vitro anti-cancer studies, and in silico investigations
Source: PLoS One. 2025 Aug 26;20(8):e0328221. doi: 10.1371/journal.pone.0328221 (PMC12380347; doi:10.1371/journal.pone.0328221)
Supplement: S1 File — Fig. S1. Molecular docking results of all compounds. Fig. S2. Toxicity prediction results. Fig. S3 FTIR, MS and NMR spectra of all compounds. Table S1. In silico hepatoxicity prediction results. Table S2 and Table S3. Green metric calculations. (DOCX) [file pone.0328221.s001.docx]

**Novel Thiazole Derivatives As Effective Anti-Cancer Agents Against Human Osteosarcoma Cell Line (SaOS-2): Microwave-Assisted One-Pot Three-Component Synthesis, In Vitro Anti-cancer Studies, And In Silico Investigations**

**Materials and methods**

All the chemicals were purchased from Merck and used without further purification. Reactions were monitored by thin-layer chromatography (TLC) on silica gel 60 F254 aluminum sheets and detection was made using UV light. FT-IR spectra were recorded using a Perkin Elmer 1600 series FTIR spectrometer. ^1^H NMR and ^13^C NMR spectra were registered on a BRUKER AVENE II 400 MHz NMR spectrometer. The chemical shifts are given in ppm relative to Me4Si as an internal reference, *J* values are given in Hz. The mass spectra were obtained on a Quattro EI-MS (70 eV) Instrument. Microwave irradiated reactions were performed in a CEM Discovery mono-mode synthesis reactor.

**Fig. S1.** Predicted binding modes and key interactions of all synthesis compounds (4a-4j) in EGFR active site from molecular docking and MM-GBSA analysis.


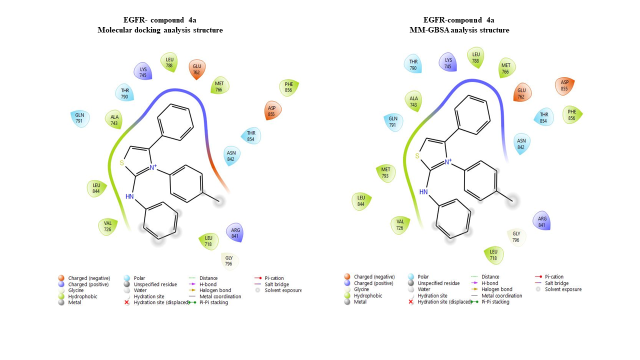


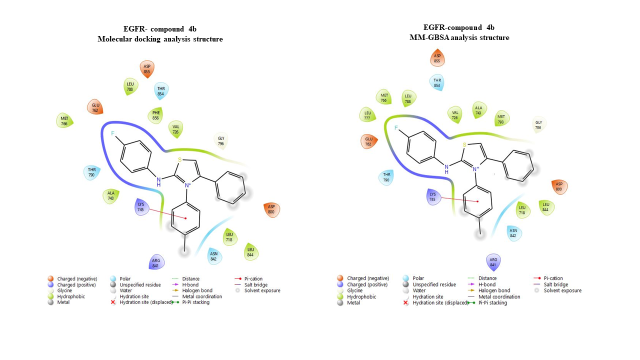


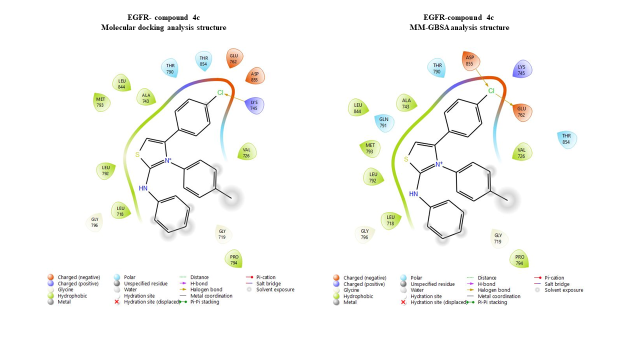


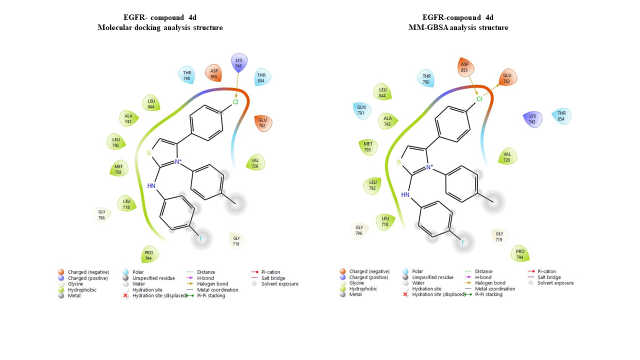


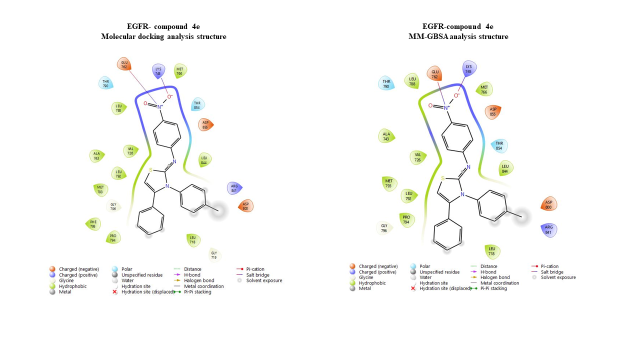


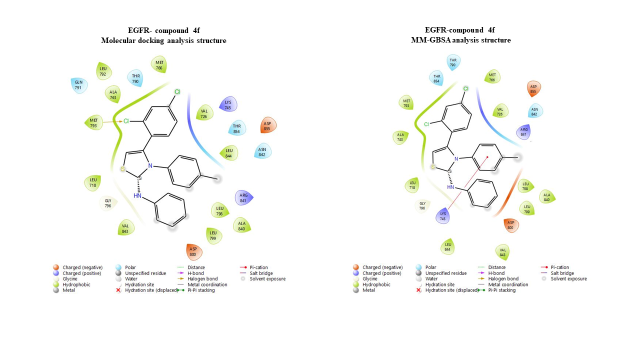


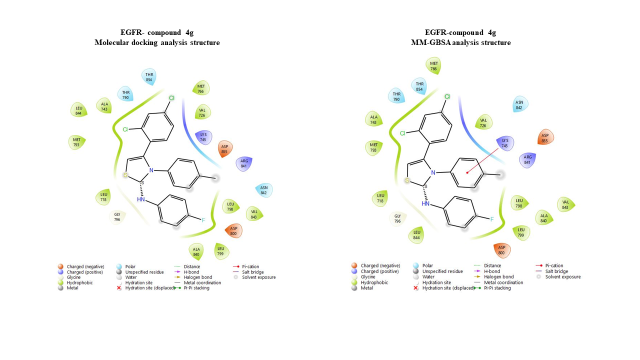


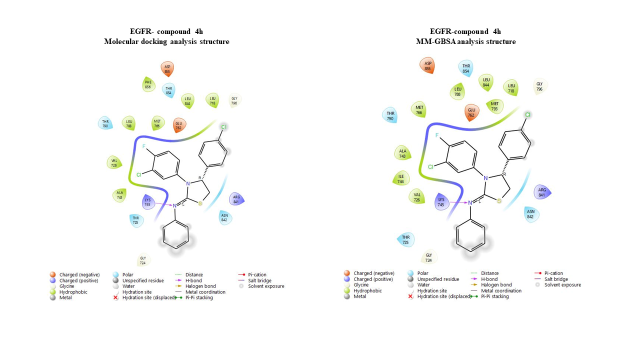


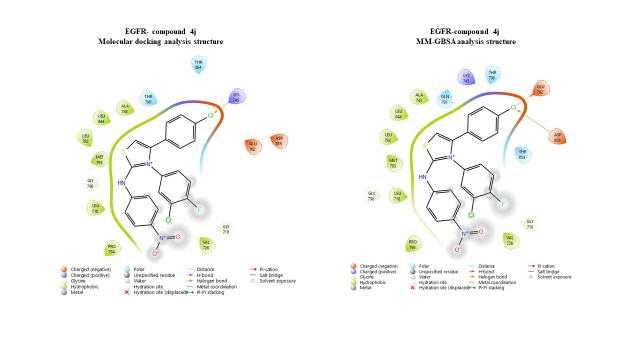


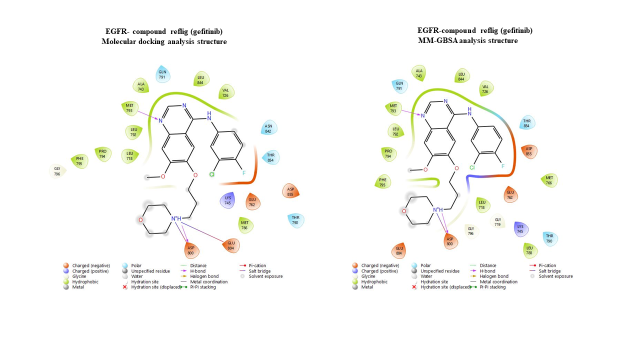


**Figure S2**. In silico toxicity prediction results using ProTox-3.0


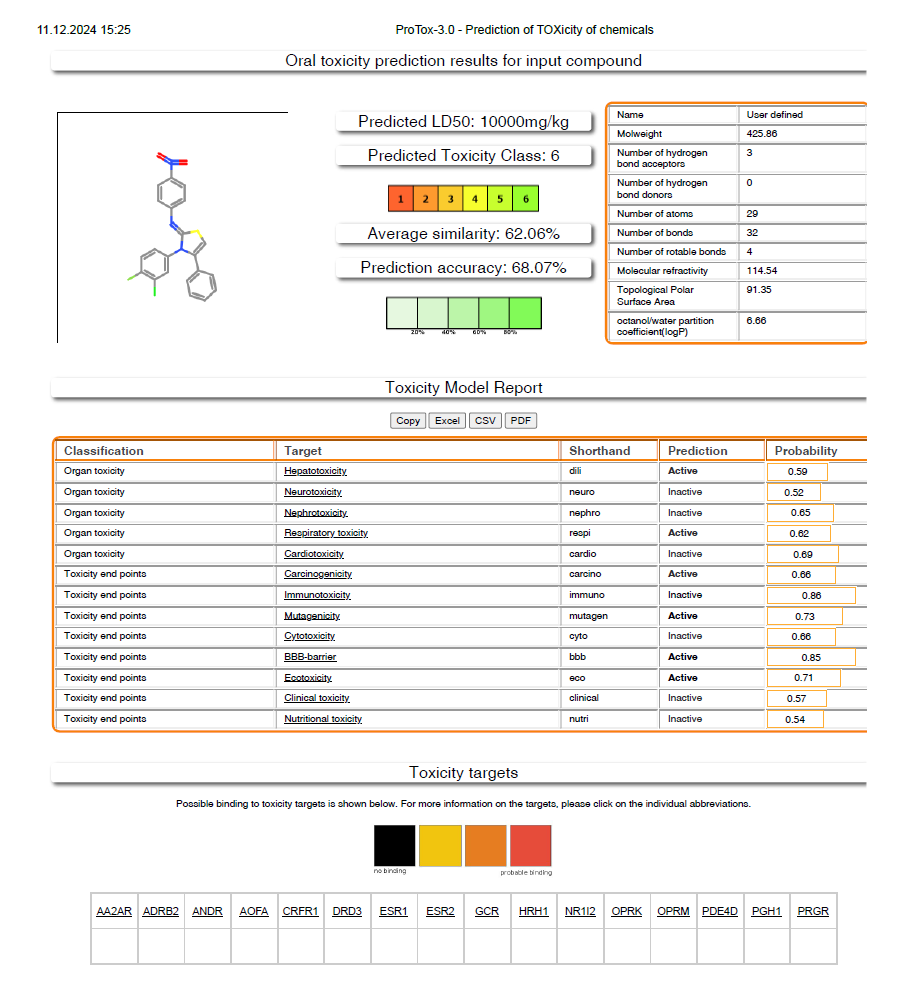


**Table S1.** In silico hepatoxicity prediction results using pkCSM

|  | **hERGI** | **Hepatotoxicity** |
| --- | --- | --- |
| **Reflig (gefitinib)** | No | Yes |
| **4a** | No | Yes |
| **4b** | No | Yes |
| **4c** | No | No |
| **4d** | No | Yes |
| **4e** | No | No |
| **4f** | No | No |
| **4g** | No | Yes |
| **4h** | No | No |
| **4i** | No | Yes |
| **4j** | No | No |

**Figure S3.** Spectral characterization of the synthesized compounds


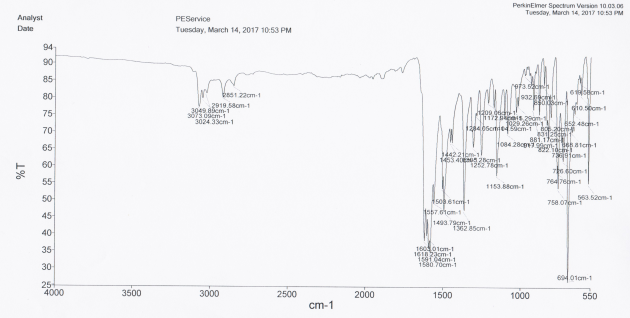


FT-IR spectra of compound **4a**


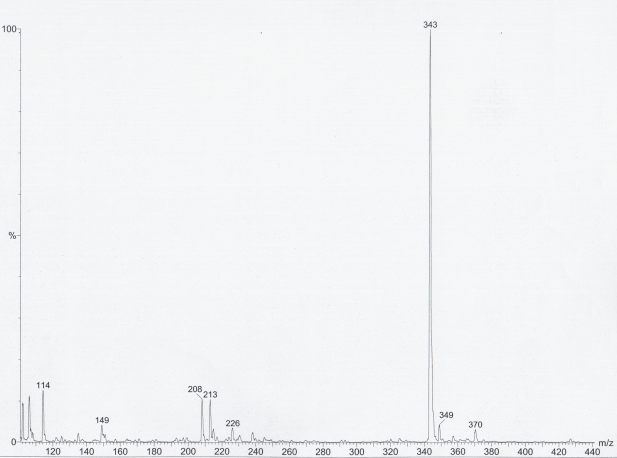


EI-MS spectra of compound **4a**


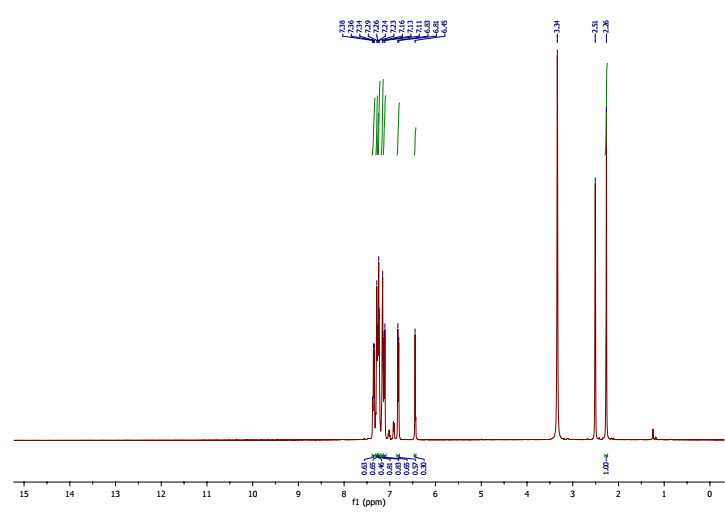


^1^H NMR spectra of compound **4a**


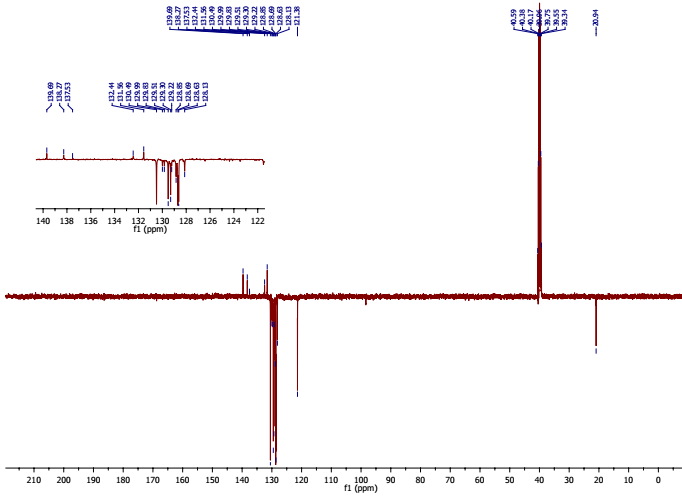


^13^C NMR (APT) spectra of compound **4a**


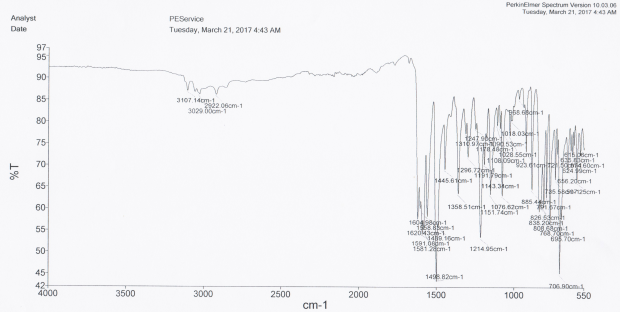


FT-IR spectra of compound **4b**


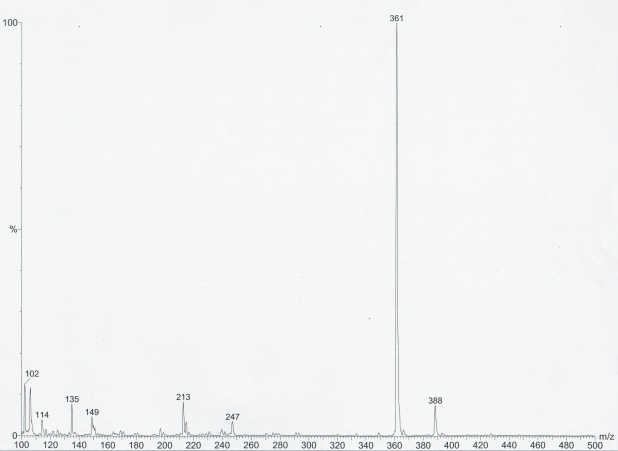


EI-MS spectra of compound **4b**


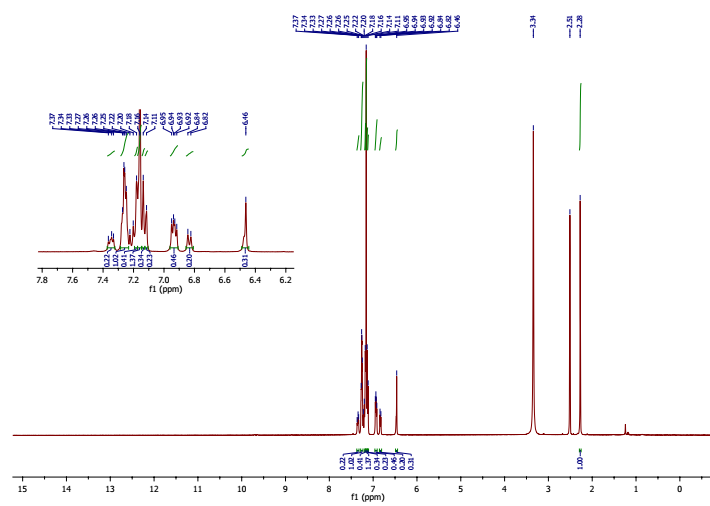


^1^H NMR spectra of compound **4b**


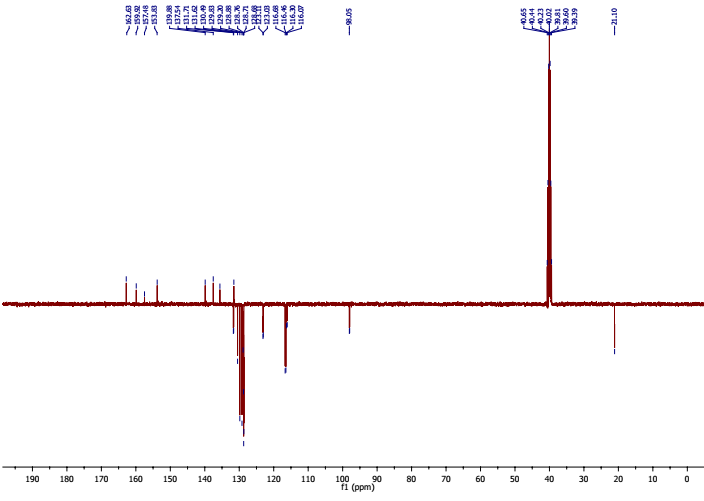


^13^C NMR (APT) spectra of compound **4b**


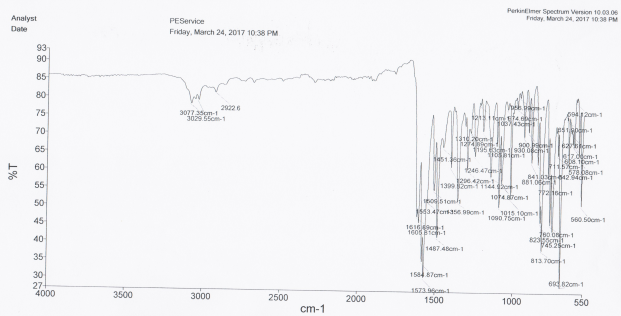


FT-IR spectra of compound **4c**


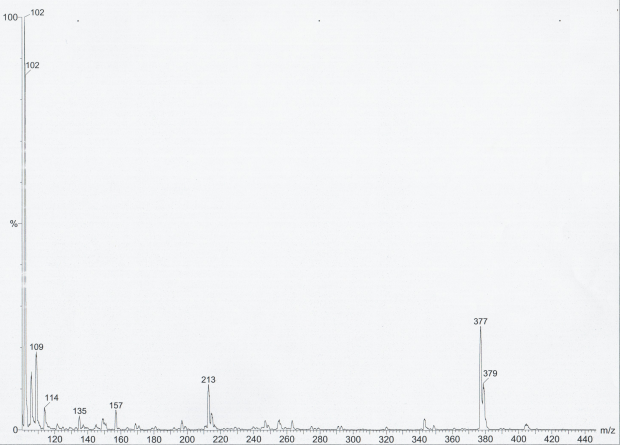


EI-MS spectra of compound **4c**


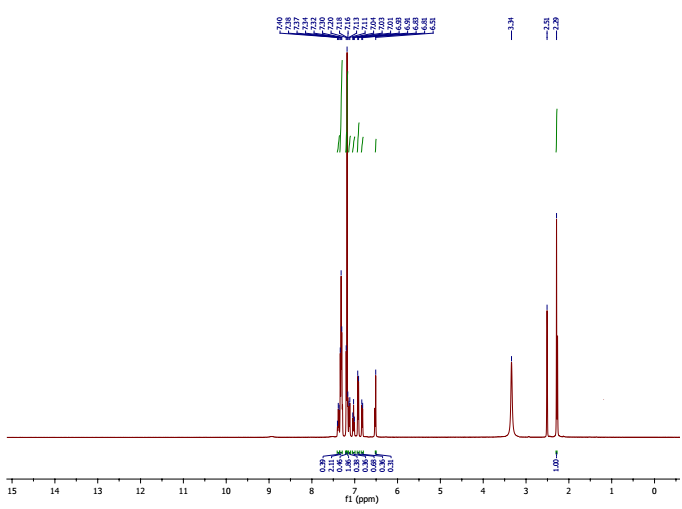


^1^H NMR spectra of compound **4c**


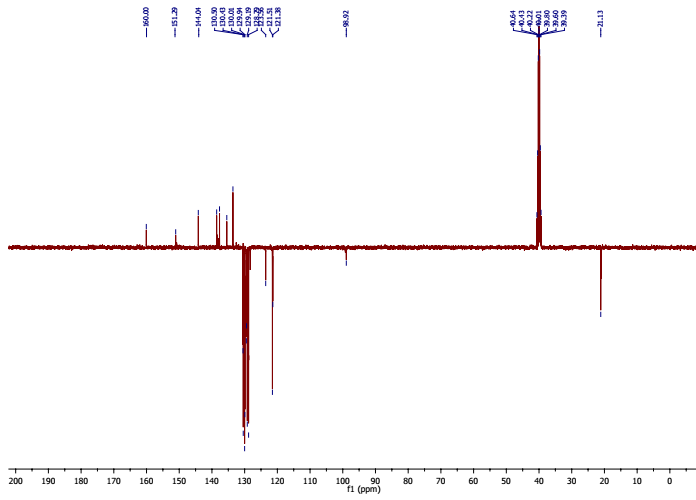


^13^C NMR (APT) spectra of compound **4c**


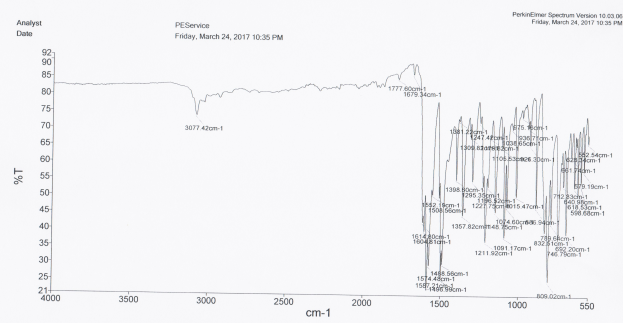


FT-IR spectra of compound **4d**


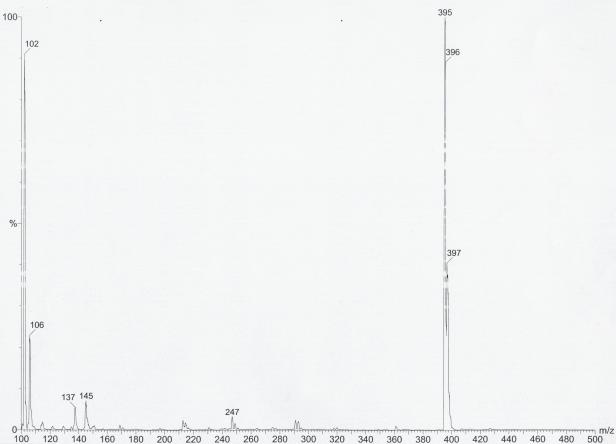


EI-MS spectra of compound **4d**


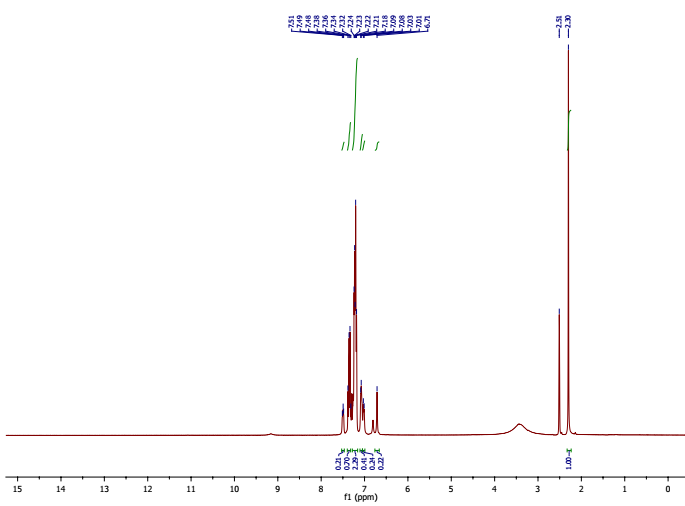


^1^H NMR spectra of compound **4d**


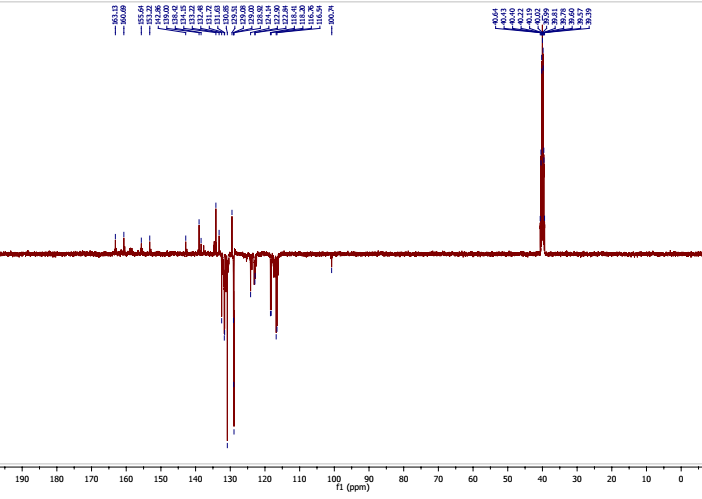


^13^C NMR (APT) spectra of compound **4d**


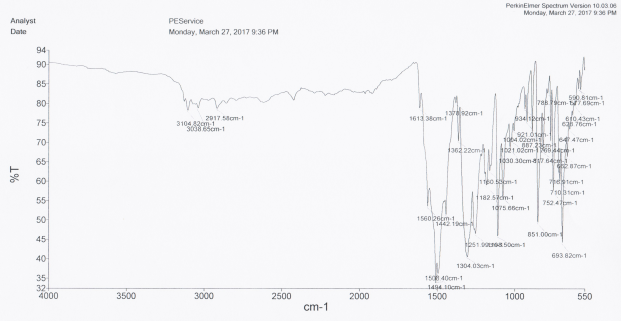


FT-IR spectra of compound **4e**


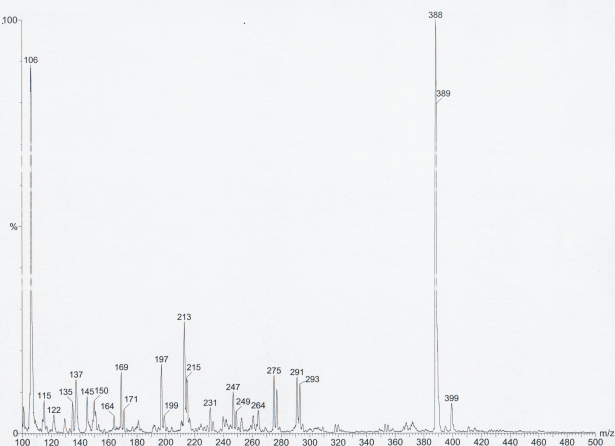


EI-MS spectra of compound **4e**


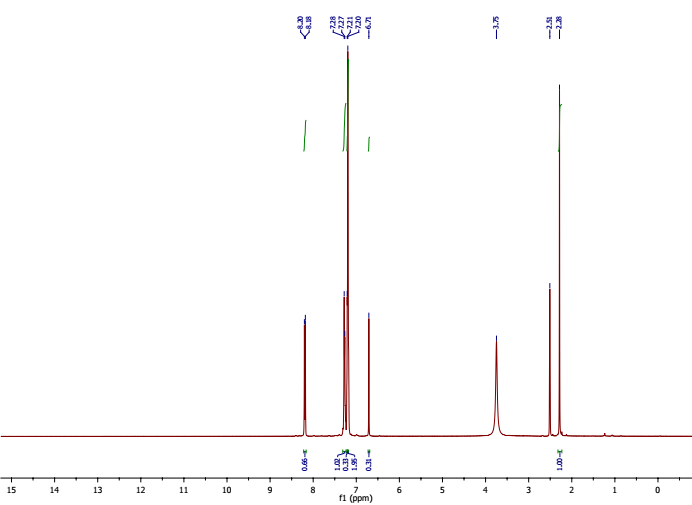


^1^H NMR spectra of compound **4e**


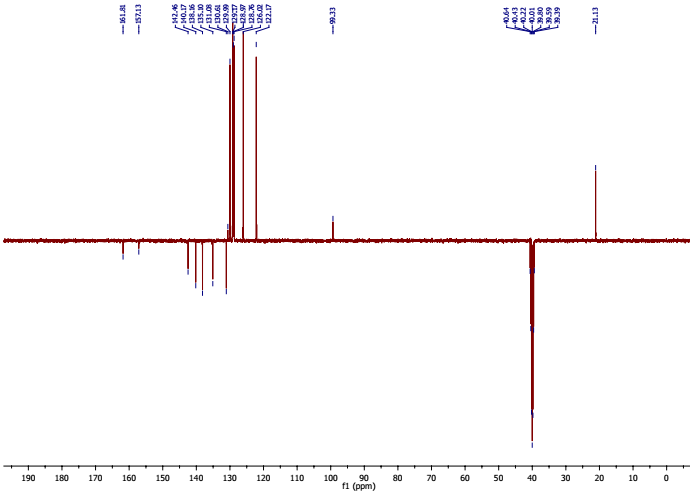


^13^C NMR (APT) spectra of compound **4e**


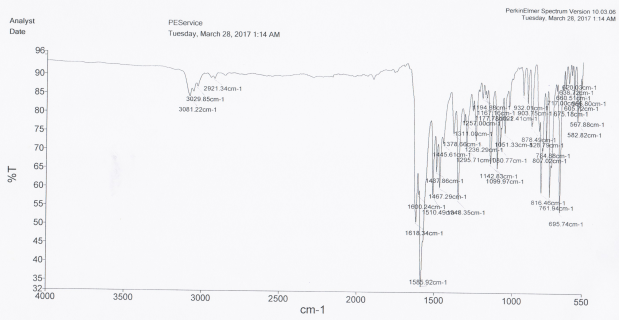


FT-IR spectra of compound **4f**


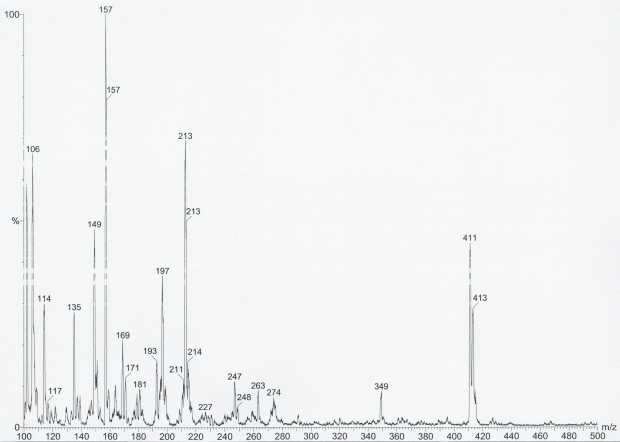


EI-MS spectra of compound **4f**


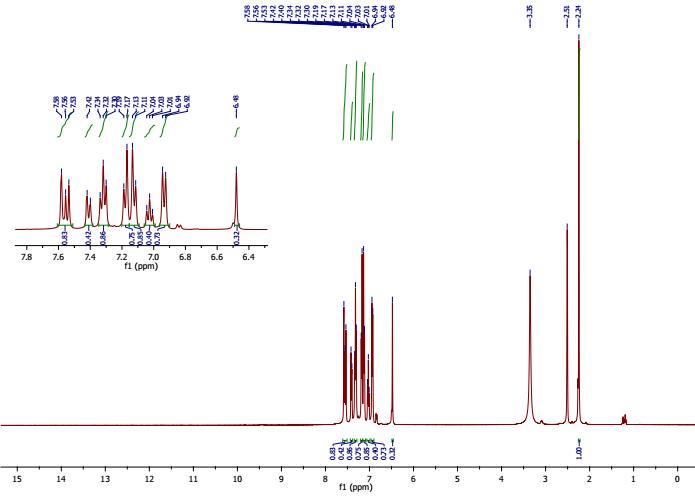


^1^H NMR spectra of compound **4f**


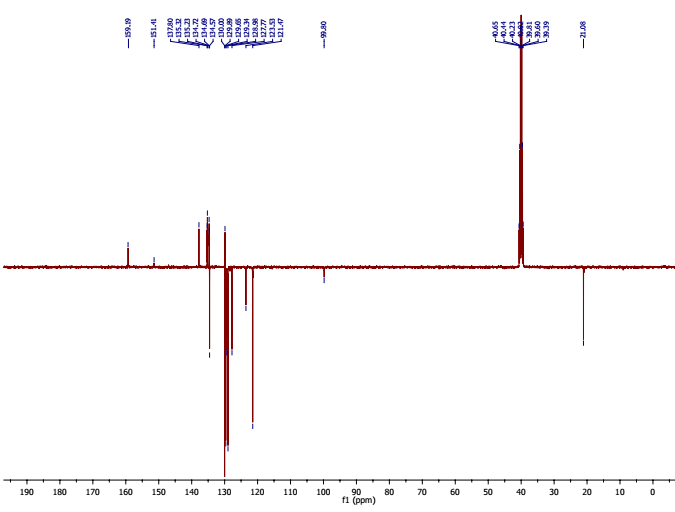


^13^C NMR (APT) spectra of compound **4f**


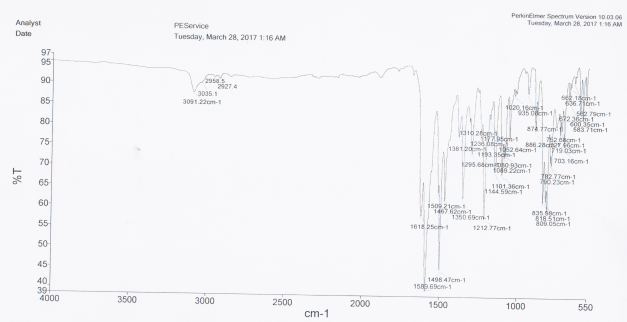


FT-IR spectra of compound **4g**


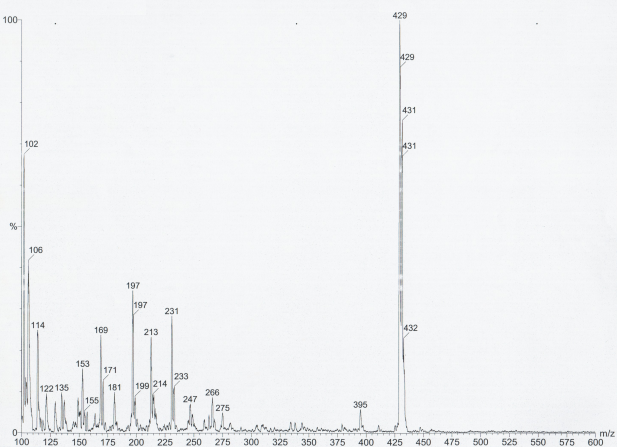


EI-MS spectra of compound **4g**


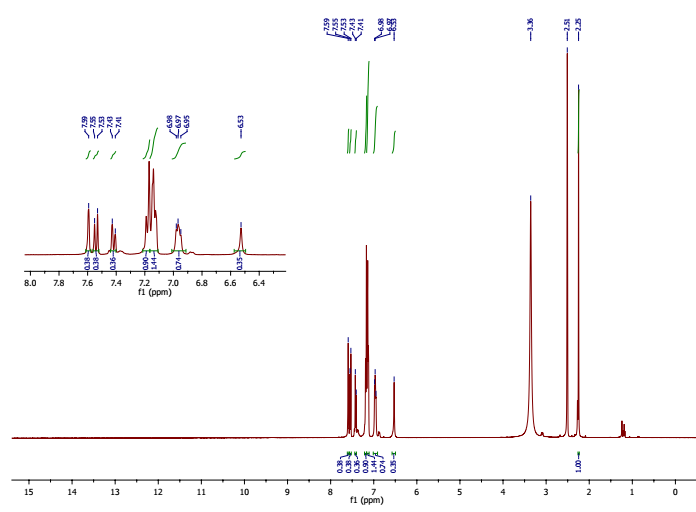


^1^H NMR spectra of compound **4g**


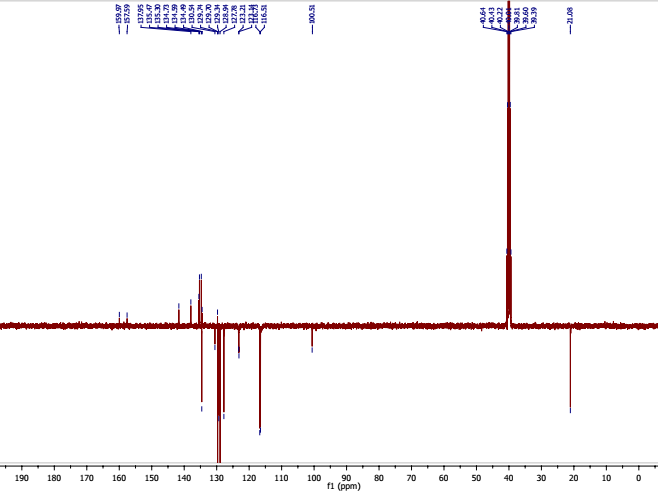


^13^C NMR (APT) spectra of compound **4g**


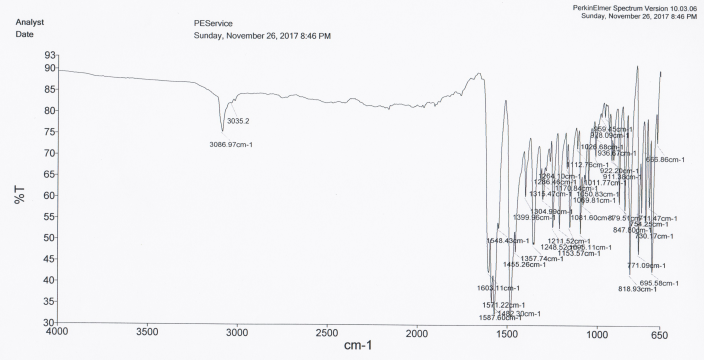


FT-IR spectra of compound **4h**


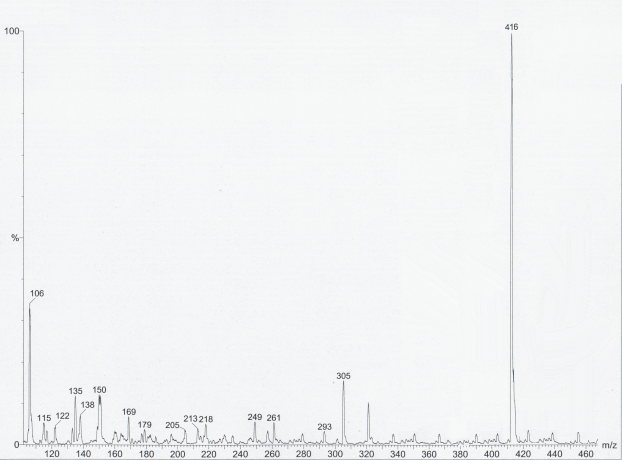


EI-MS spectra of compound **4h**


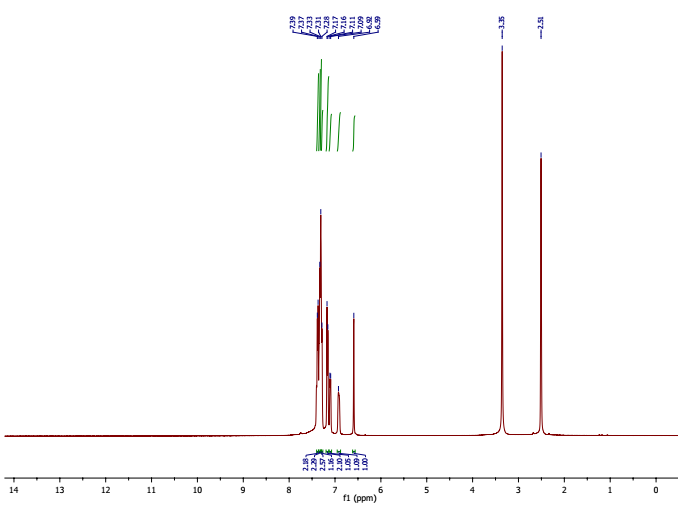


^1^H NMR spectra of compound **4h**


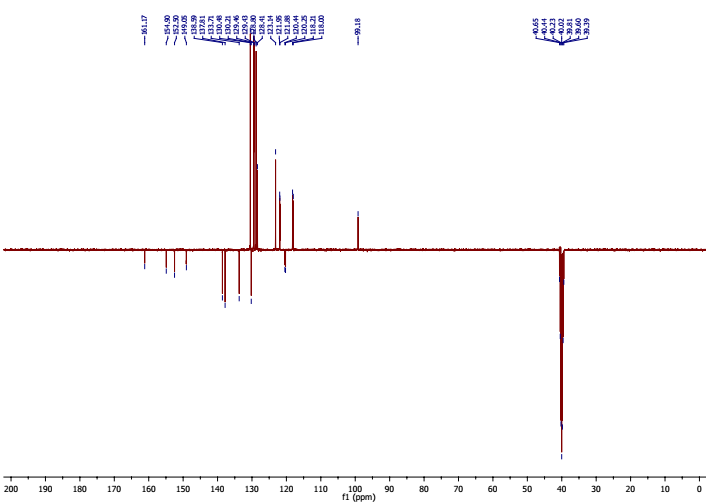


^13^C NMR (APT) spectra of compound **4h**


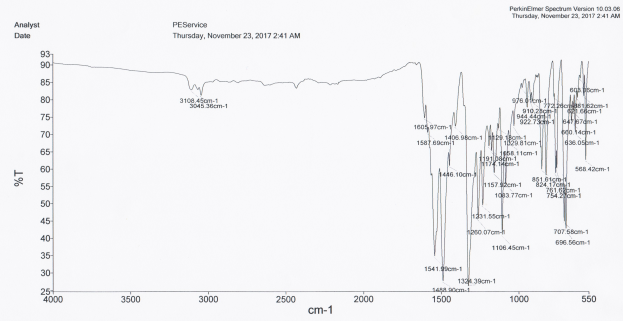


FT-IR spectra of compound **4i**


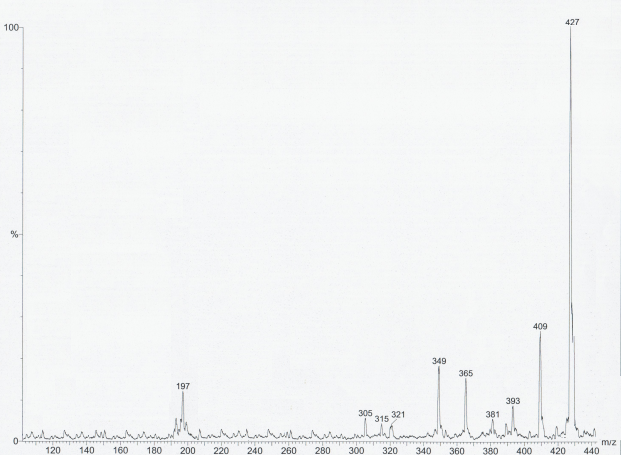


EI-MS spectra of compound **4i**


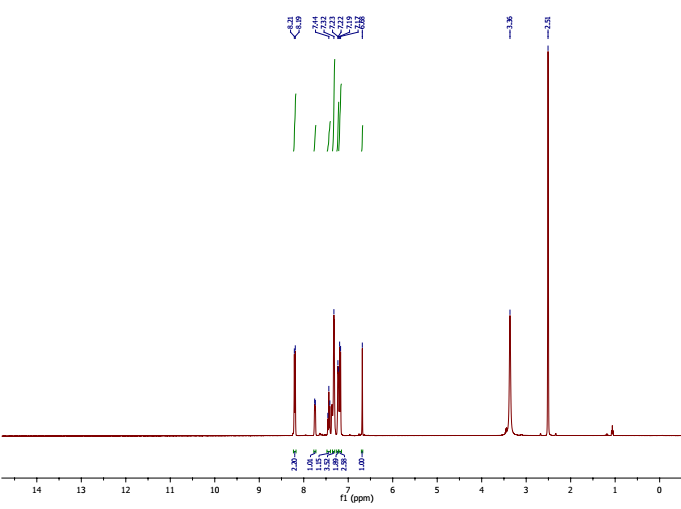


^1^H NMR spectra of compound **4i**


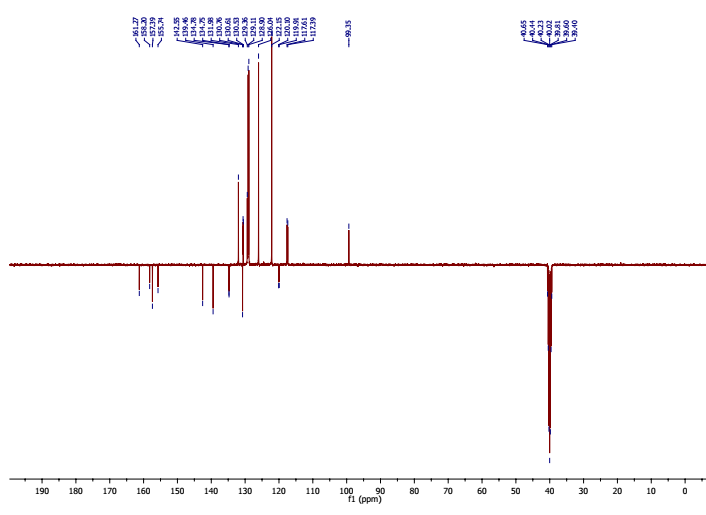


^13^C NMR (APT) spectra of compound **4i**


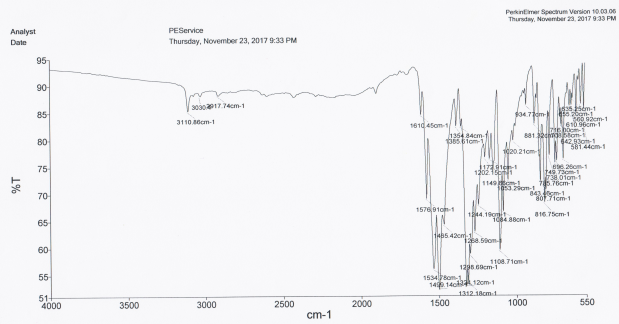


FT-IR spectra of compound **4j**


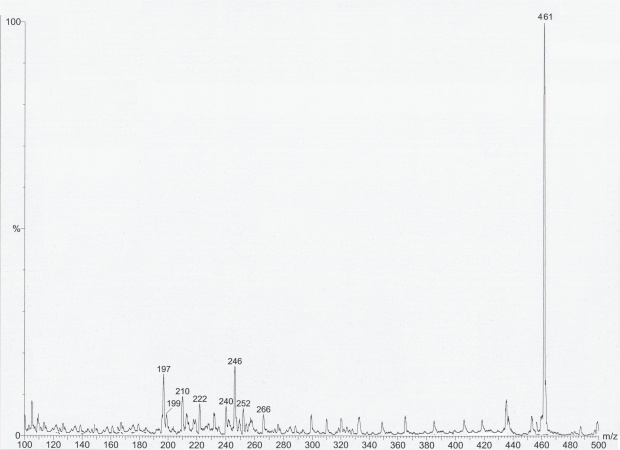


EI-MS spectra of compound **4j**


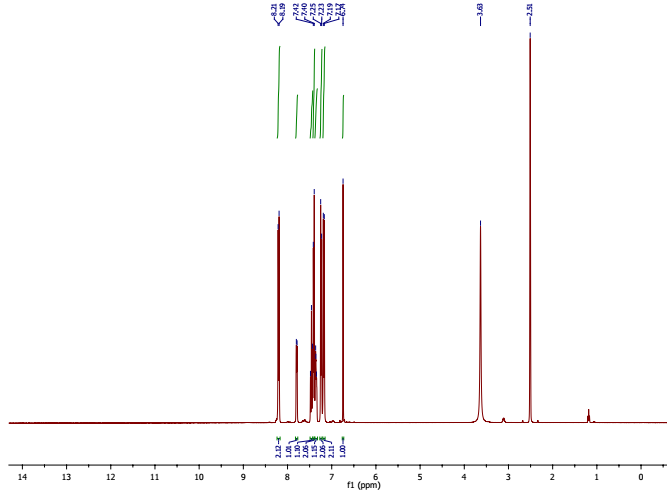


^1^H NMR spectra of compound **4j**


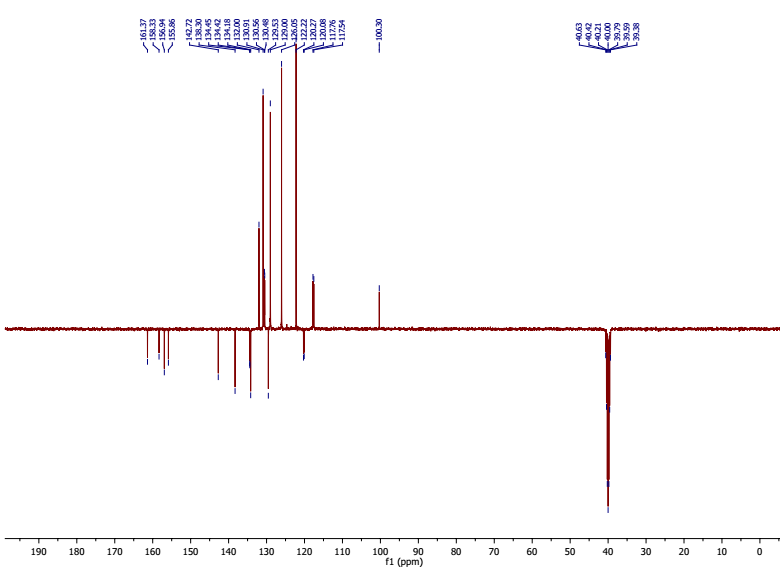


^13^C NMR (APT) spectra of compound **4j**

**Table S2. Green Metric Calculations**

| **Comp.** | Mol. Weight | Yield (MW) | Yield (CM) | Formula | Mass |
| --- | --- | --- | --- | --- | --- |
| **4a** | 342.456 | 89 (0.304 g) | 79 (0.269) | C_22_H_18_N_2_S | 0,342 |
| **4b** | 360.109 | 86 (0.3096) | 73 (0.262) | C_22_H_17_FN_2_S | 0,36 |
| **4c** | 376.080 | 92 (0.346) | 77 (0.289) | C_22_H_17_ClN_2_S | 0,376 |
| **4d** | 394.070 | 88 (0.347) | 74 (0.291) | C_22_H_16_ClFN_2_S | 0,394 |
| **4e** | 387.104 | 82 (0.317) | 69 (0.266) | C_22_H_17_N_3_SO_2_ | 0,387 |
| **4f** | 410.041 | 80 (0.328) | 71 (0.291) | C_22_H_16_Cl_2_N_2_S | 0,41 |
| **4g** | 428.031 | 81 (0.347) | 70 (0.299) | C_22_H_15_Cl_2_FN_2_S | 0,428 |
| **4h** | 414.016 | 84 (0.348) | 73 (0.302) | C_21_H_13_Cl_2_FN_2_S | 0,414 |
| **4i** | 425.040 | 79 (0.336) | 68 (0.289) | C_21_H_13_ClFN_3_O_2_S | 0,425 |
| **4j** | 460.308 | 82 (0.377) | 70 (0.321) | C_21_H_12_Cl_2_FN_3_O_2_S | 0,46 |

**Table S3. Green Metric Calculations**

| **Comp.** | MI-1 | MI-2 | RME-1 | RME-2 | CE-1 | CE-2 | AE-1 | AE-2 |
| --- | --- | --- | --- | --- | --- | --- | --- | --- |
| **4a** | 30,89 | 9,263 | 58 | 66 | 79 | 89 | 78 | 78 |
| **4b** | 31,79 | 9,1549 | 55 | 65 | 73 | 86 | 79 | 79 |
| **4c** | 28,87 | 8,237 | 58 | 70 | 77 | 92 | 79 | 79 |
| **4d** | 28,74 | 8,265 | 57 | 67 | 74 | 88 | 80 | 80 |
| **4e** | 31,33 | 9,13 | 53 | 63 | 69 | 82 | 80 | 80 |
| **4f** | 28,79 | 8,79 | 55 | 62 | 71 | 80 | 80 | 80 |
| **4g** | 28,08 | 8,36 | 54 | 63 | 70 | 81 | 81 | 81 |
| **4h** | 27,75 | 8,27 | 56 | 65 | 73 | 84 | 80 | 80 |
| **4i** | 29,04 | 8,62 | 53 | 62 | 68 | 79 | 81 | 81 |
| **4j** | 26,25 | 7,78 | 55 | 65 | 70 | 82 | 82 | 82 |

E- factor, MI mass intensity, % RME percentage reaction mass efficiency, % CE percentage carbon efficiency, % AE percentage atom economy. 1: Conventional method; 2: Microwave Irradiation method.
